# Supplementary material for: Discovery of Oleaginous Yeast from Mountain Forest Soil in Thailand
Source: J Fungi (Basel). 2022 Oct 18;8(10):1100. doi: 10.3390/jof8101100 (PMC9605381; doi:10.3390/jof8101100)
Supplement: Supplementary file 1 [file jof-08-01100-s001.zip › jof-1937450-supplementary data S1.pdf]

Supplementary Data

Table S1. Sampling location, physical properties of soil in the sampling sites and yeast isolated by NDM-agar and NDM-gellan gum.

Sampling location	Soil conditions			Number of strains		Total of strains
	Temp. (C°)	Humidity (%)	pH	NDM-agar	NDM-gellan gum	
Sample 1	20	15	6.5	1	-	1
Sample 2	21	15	7.0	1	1	2
Sample 3	22	15	7.0	1	-	1
Sample 4	15	18	7.0	1	-	1
Sample 5	18	20	7.0	1	6	7
Sample 6	20	30	6.5	3	5	8
Sample 7	25	15	7.0	1	5	6
Sample 8	18	30	7.5	4	2	6
Sample 9	18	20	7.0	12	12	24
Sample 10	19	30	8.5	-	5	5
Sample 11	19	30	4.0	3	1	4
Sample 12	19	45	6.0	2	1	3
Sample 13	19	30	6.5	1	1	2
Sample 14	18	40	7.0	-	3	3
Sample 15	19	40	7.0	1	-	1
Sample 16	19	30	6.5	4	-	4
Sample 17	20	20	7.0	1	8	9
Sample 18	19	45	6.5	2	-	2
Sample 19	18	15	7.0	4	1	5
Sample 20	19	15	7.0	2	1	3
Sample 21	18	15	6.5	11	13	24
Sample 22	15	19	7.0	-	6	6
Total				56	71	127

Table S2. Lipid accumulation of yeast isolated cultivated in 50 mL of 2G2M broth containing 20 g L^{-1} glucose on rotary shaker at 150 rpm and 30°C for 7 d.

Yeast species	Strain	Biomass (g L^{-1})	Lipid (g L^{-1})	Lipid content (% of dry biomass)
Lipomyces tetrasporus	SWU-NGP 2-5	1.49±0.05	1.10±0.06	74.26±6.30
Lipomyces tetrasporus	SWU-NAP 4-1	4.50±0.08	2.57±0.04	57.10±1.82
Lipomyces mesembrius	SWU-NGP 14-6	9.13±0.36	5.20±0.03	57.10±1.89
Lipomyces mesembrius	SWU-NGP 6-4	7.83±0.17	3.98±0.02	50.80±0.80
Lipomyces tetrasporus	SWU-NGP 5-8-1	9.07±0.16	4.27±0.01	47.06±0.66
Saitozyma podzolica	SWU-NAP 5-4-2	3.88±0.19	1.81±0.19	46.63±2.51
Lipomyces starkeyi	SWU-NGP 14-3-1	3.53±0.56	1.47±0.07	42.49±4.65
Lipomyces starkeyi	SWU-NGTP 4-5	5.92±0.14	2.45±0.22	41.56±4.67
Lipomyces starkeyi	SWU-NGP 5-7	8.39±0.85	3.35±0.002	40.30±4.11
Cyberlindnera saturnus	SWU-NGTP 5-3-3	1.00±0.04	0.4±0.014	40.00±0.00
Saitozyma podzolica	SWU-NGP 5-3-8	6.59±0.89	2.63±0.35	40.00±0.00
Papiliotrema flavescens	SWU-NGTP 4-1	1.87±0.24	0.74±0.10	40.00±0.00
Saitozyma podzolica	SWU-NGP 14-2-2	4.99±0.19	1.99±0.07	40.00±0.00
Meyerozyma guilliermondii	SWU-NATP 2-4	0.25±0.04	0.10±0.02	40.00±0.00
Papiliotrema flavescens	SWU-NATP 3-3	1.15±0.06	0.46±0.02	40.00±0.00
Lipomyces starkeyi	SWU-NGTP 4-3	6.03±0.15	2.40±0.18	39.69±2.00
Papiliotrema terrestris	SWU-NAP 12-1-1	3.46±0.04	1.34±0.25	38.76±7.80
Lipomyces mesembrius	SWU-NAP 3-4	8.40±0.20	3.25±0.33	38.64±3.03
Lipomyces starkeyi	SWU-NATP 4-2	2.93±0.09	1.13±0.13	38.60±5.62
Saitozyma podzolica	SWU-NGP 5-3-2-1	2.42±0.02	0.92±0.05	38.00±1.89
Lipomyces mesembrius	SWU-NAP 8-4	7.88±0.02	2.86±0.12	36.32±1.59
Lipomyces mesembrius	SWU-NGP 14-8	9.87±0.05	3.54±0.10	35.92±0.85
Lipomyces starkeyi	SWU-NGTP 5-7	9.50±0.46	3.38±0.09	35.71±2.66
Lipomyces starkeyi	SWU-NGP 6-6	4.88±0.11	1.71±0.13	35.04±3.45
Lipomyces mesembrius	SWU-NATP 1-4	2.42±0.01	0.84±0.29	34.68±11.77
Lipomyces tetrasporus	SWU-NAP 9-2	8.82±0.17	2.95±0.54	33.32±5.48
Papiliotrema terrestris	SWU-NAPui 12-9	5.99±0.38	1.96±0.36	32.57±3.97
Lipomyces tetrasporus	SWU-NAP 13-8	7.60±0.90	2.39±0.15	32.14±5.83
Lipomyces starkeyi	SWU-NATP 2-6	5.22±0.15	1.66±0.08	31.91±2.41
Saitozyma podzolica	SWU-NGP 5-3-2	7.35±0.13	2.33±0.11	31.71±0.90
Lipomyces starkeyi	SWU-NAP 13-4	9.93±0.22	3.12±0.07	31.47±1.36
Lipomyces tetrasporus	SWU-NAP 5-2-2	2.76±0.21	0.86±0.14	30.94±2.71
Lipomyces tetrasporus	SWU-NAP 5-3-1-1	8.69±0.85	2.76±1.26	30.59±11.51
Saitozyma podzolica	SWU-YGP 8-1-2	3.30±0.00	0.97±0.19	29.33±5.81
Lipomyces tetrasporus	SWU-NATP 2-12	3.58±0.42	1.01±0.15	28.98±7.48
Lipomyces starkeyi	SWU-NGP 14-9	6.27±0.04	1.80±0.01	28.68±0.09
Lipomyces starkeyi	SWU-NGTP 4-8	8.42±0.24	2.38±0.02	28.23±0.52
Papiliotrema terrestris	SWU-NAP 12-1-12	7.12±0.48	2.01±0.34	28.06±2.88
Lipomyces starkeyi	SWU-NAP 2-2	2.12±0.15	0.59±0.03	27.93±0.50
Lipomyces tetrasporus	SWU-NAP 5-2-1	7.34±0.22	2.04±0.71	27.61±8.90
Lipomyces starkeyi	SWU-NGTP 4-11	6.63±0.57	1.78±0.09	27.22±3.67

Table S2. *Cont.*

Yeast species	Strain	Biomass (g L^{-1})	Lipid (g L^{-1})	Lipid content (% of dry biomass)
Lipomyces tetrasporus	SWU-NGP 6-5	3.47±0.17	0.93±0.01	26.94±1.55
Lipomyces tetrasporus	SWU-NGP 9-3	10.15±0.20	2.73±0.21	26.93±2.62
Lipomyces tetrasporus	SWU-NGP 11-1	10.95±0.06	2.90±0.13	26.45±1.31
Lipomyces tetrasporus	SWU-NGP 3-7	8.89±0.11	2.35±0.14	26.44±1.25
Lipomyces tetrasporus	SWU-NGP 4-2-2	9.37±0.86	2.44±0.17	26.42±4.27
Lipomyces tetrasporus	SWU-NGP 2-1-1	5.87±0.04	1.54±0.11	26.27±2.07
Lipomyces tetrasporus	SWU-NAP 2-1	8.87±0.10	2.30±0.08	25.88±0.66
Lipomyces starkeyi	SWU-NAPui 2-1	1.35±0.08	0.35±0.04	25.83±1.58
Piskurozyma sp.	SWU-NATP 4-12	2.92±0.24	0.74±0.04	25.45±0.82
Lipomyces starkeyi	SWU-NGTP 4-4	7.16±1.26	1.81±0.28	25.42±0.62
Lipomyces tetrasporus	SWU-NGP 3-6	8.81±0.10	2.21±0.08	25.16±1.20
Saitozyma podzolica	SWU-NGP 5-10-2	1.71±0.12	0.42±0.01	24.54±2.12
Lipomyces starkeyi	SWU-NAP 8-1	8.60±0.58	2.11±0.20	24.49±0.65
Lipomyces mesembrius	SWU-NATP 4-13	2.72±0.19	0.66±0.05	24.15±0.12
Lipomyces starkeyi	SWU-NATP 4-5	6.78±0.11	1.60±0.13	23.62±1.53
Papiliotrema flavescens	SWU-YGPui 12-1	5.81±1.51	1.25±0.06	23.43±7.18
Lipomyces mesembrius	SWU-NATP 4-11	3.59±0.25	0.83±0.02	23.32±2.07
Cyberlindnera saturnus	SWU-NATP 4-4	3.65±0.55	0.81±0.07	22.94±5.32
Papiliotrema flavescens	SWU-NAP1-4	8.11±0.10	1.85±0.08	22.86±1.21
Lipomyces tetrasporus	SWU-NGP 2-6-2	2.31±0.14	0.53±0.05	22.86±0.74
Lipomyces starkeyi	SWU-NAPH 3-6	3.93±0.31	0.9±0.018	22.62±2.73
Lipomyces tetrasporus	SWU-NATP 9-2	7.57±0.06	1.69±0.19	22.38±2.35
Lipomyces mesembrius	SWU-NAP 8-2	5.37±0.15	1.19±0.01	22.13±0.86
Lipomyces tetrasporus	SWU-NAP 5-5	2.86±0.17	0.62±0.01	21.87±1.47
Lipomyces tetrasporus	SWU-NGP 2-2	6.15±0.75	1.33±0.12	21.77±0.75
Hannaella sp.	SWU-YGP 11-1	1.70±0.15	0.36±0.08	21.69±6.69
Lipomyces tetrasporus	SWU-NGP 2-3	7.89±0.05	1.70±0.04	21.54±0.42
Lipomyces starkeyi	SWU-NGP 5-3-3	6.52±0.17	1.39±0.16	21.44±3.00
Lipomyces tetrasporus	SWU-NAP 6-1	6.15±0.64	1.31±0.14	21.28±0.08
Lipomyces tetrasporus	SWU-NAP 5-6	3.24±0.92	0.66±0.09	21.18±2.62
Lipomyces tetrasporus	SWU-NGP 1-3	8.90±0.91	1.85±0.18	20.85±0.13
Papiliotrema flavescens	SWU-NGTP 3-2	3.45±0.05	0.72±0.03	20.83±0.71
Lipomyces tetrasporus	SWU-NGTP 4-2	2.10±0.06	0.43±0.03	20.65±0.65
Lipomyces tetrasporus	SWU-NAP 4-2-1	8.27±0.32	1.68±0.64	20.60±8.54
Lipomyces starkeyi	SWU-NGTP 4-12-1	5.29±0.04	1.08±0.19	20.48±3.82
Lipomyces tetrasporus	SWU-NAP 2-3	7.89±0.14	1.60±0.02	20.23±0.16
Cyberlindnera saturnus	SWU-NGTP 4-12-2	1.98±0.16	0.40±0.01	20.13±0.97
Cyberlindnera saturnus	SWU-NGTP 4-14-2	1.76±0.06	0.35±0.06	19.92±4.32
Lipomyces tetrasporus	SWU-NAP 5-3-3	5.46±0.34	1.07±0.16	19.87±4.19
Lipomyces tetrasporus	SWU-NAP 5-4	7.44±0.00	1.46±0.03	19.65±0.40
Lipomyces tetrasporus	SWU-NGP 5-9-2	8.19±0.25	1.59±0.05	19.40±1.15
Lipomyces mesembrius	SWU-NATP 4-8	6.63±0.62	1.26±0.03	19.07±1.30

Table S2. *Cont.*

Yeast species	Strain	Biomass (g L^{-1})	Lipid (g L^{-1})	Lipid content (% of dry biomass)
Lipomyces tetrasporus	SWU-NAP 5-4-1	9.56±0.22	1.80±0.18	18.84±1.45
Lipomyces starkeyi	SWU-NATP 1-1	5.82±0.80	1.08±0.21	18.36±1.15
Lipomyces starkeyi	SWU-NAP 14-4	2.28±0.13	0.40±0.07	17.87±4.09
Lipomyces starkeyi	SWU-NATP 4-3	5.45±0.26	0.95±0.10	17.61±2.66
Lipomyces tetrasporus	SWU-NGP 4-14	9.48±0.24	1.66±0.15	17.55±1.98
Lipomyces tetrasporus	SWU-NGP 10-3	8.37±0.12	1.46±0.04	17.44±0.69
Lipomyces tetrasporus	SWU-NGP 11-2	9.33±0.03	1.62±0.10	17.39±1.11
Papiliotrema flavescens	SWU-NAP 3-2	1.66±0.50	0.29±0.09	17.32±0.08
Lipomyces starkeyi	SWU-NGP 5-10-8	6.75±0.12	1.16±0.20	17.15±2.73
Lipomyces tetrasporus	SWU-NGP 5-3-1	8.82±0.02	1.49±0.01	16.95±0.18
Lipomyces starkeyi	SWU-NGP 1-5-4	0.9±0.11	0.15±0.03	16.85±1.15
Papiliotrema flavescens	SWU-NATP 2-7	6.23±0.28	1.03±0.02	16.63±1.12
Hannaella sp.	SWU-NAPS 5-1	6.41±0.65	1.06±0.15	16.46±0.72
Lipomyces starkeyi	SWU-NGTP 4-12-3	4.83±0.83	0.79±0.14	16.40±0.002
Lipomyces tetrasporus	SWU-NGP 1-1	8.44±0.06	1.38±0.09	16.32±1.00
Lipomyces starkeyi	SWU-NAP 10-1	3.52±0.27	0.58±0.09	16.31±1.42
Lipomyces starkeyi	SWU-NATP 3-1	4.63±0.04	0.74±0.02	15.92±0.55
Lipomyces tetrasporus	SWU-NGP 3-1	6.82±0.05	1.07±0.01	15.63±0.03
Papiliotrema flavescens	SWU-NGTP 2-6	6.00±0.74	0.86±0.04	14.47±.1.12
Piskurozyma sp.	SWU-NGP 14-3-2	0.53±0.15	0.07±0.01	14.12±5.88
Lipomyces tetrasporus	SWU-NAP 5-1-2	3.46±0.44	0.49±0.13	14.00±1.94
Lipomyces tetrasporus	SWU-NGP 5-6-1	5.35±0.34	0.73±0.05	13.85±1.88
Papiliotrema terrestris	SWU-NAPui 14-5	8.25±1.15	1.15±0.28	13.70±1.53
Rhodotorula mucilaginosa	SWU-NGP 14-3-3	6.04±0.11	0.82±0.06	13.64±0.69
Lipomyces tetrasporus	SWU-NAP 5-3-1-2	5.73±0.23	0.74±0.20	12.76±2.94
Lipomyces starkeyi	SWU-NGP 14-1	6.95±1.04	0.89±0.19	12.72±0.87
Lipomyces tetrasporus	SWU-NGP 1-4	8.51±0.03	1.07±0.05	12.58±0.67
Lipomyces starkeyi	SWU-NGTP 4-6	4.95±0.02	0.60±0.07	12.18±1.53
Piskurozyma sp.	SWU-NGP 14-3-4	0.67±0.01	0.08±0.01	11.72±0.81
Lipomyces tetrasporus	SWU-NGP 6-3	8.72±0.20	0.96±0.04	11.06±0.30
Lipomyces starkeyi	SWU-NGTP 5-8	5.52±0.01	0.60±0.14	10.88±2.62
Lipomyces tetrasporus	SWU-NGP 4-4	7.17±0.20	0.78±0.12	10.78±1.43
Papiliotrema flavescens	SWU-NATP 4-15	6.54±0.75	0.68±0.01	10.50±1.39
Saitozyma podzolica	SWU-NGP 5-4-2	5.13±0.08	0.52±0.14	10.06±2.53
Lipomyces tetrasporus	SWU-NAP 13-7	5.97±0.04	0.58±0.04	9.75±0.64
Cyberlindnera saturnus	SWU-NATP 4-6	3.70±0.20	0.34±0.23	8.81±5.85
Lipomyces tetrasporus	SWU-NGP5-4-1	2.48±0.28	0.20±0.02	8.33±1.67
Papiliotrema flavescens	SWU-NGTP 2-5	9.43±0.42	0.68±0.13	7.33±1.72
Cyberlindnera saturnus	SWU-NGP 1-5-2-1	3.08±0.02	0.22±0.003	7.30±0.01
Cyberlindnera saturnus	SWU-NGTP 4-14-3	2.73±0.01	0.19±0.01	6.81±0.19
Lipomyces tetrasporus	SWU-NGP 3-2	3.45±0.15	0.21±0.07	5.96±1.71
Lipomyces tetrasporus	SWU-NGTP 5-3-2	9.16±0.01	0.54±0.01	5.90±0.12

Table S2. *Cont.*

Yeast species	Strain	Biomass (g L^{-1})	Lipid (g L^{-1})	Lipid content (% of dry biomass)
Naganishia diffluens	SWU-NGP 1-5-1	2.72±0.00	0.15±0.01	5.64±0.00
Cystobasidium slooffiae	SWU-NAP 4-2-2	6.35±1.68	0.31±0.03	5.10±0.81
