# Supplementary material for: Discovery of Oleaginous Yeast from Mountain Forest Soil in Thailand
Source: J Fungi (Basel). 2022 Oct 18;8(10):1100. doi: 10.3390/jof8101100 (PMC9605381; doi:10.3390/jof8101100)
Supplement: Supplementary file 1 [file jof-08-01100-s001.zip › jof-1937450-supplementary data S2.pdf]

Supplementary Data 2
The D1/D2 domain of the LSU rRNA gene sequence

1. ***Lipomyces starkeyi*** **SWU-NGTP 4-8**
>SWU-NGTP 4-8 (567 bp)
AAACCAACAGGGATTGCCTTAGTAACTGCGAGTGAAGCGGCAAGAGCTCAAATT
TGAAATCTGGCACCTTCGGTGTCCGAGTTGTAATTTGTAGAAGCATCTTTTGGATT
GGTCCTTGCATATGTTCCTTGGAACAGGACATCATAGAGGGTGAGAATCCCGTTC
ATGGTGGGGAATCCAATTCTTTGTAAAGTGCTTTCGAAGAGTCGAGTTGTTTGGGA
ATGCAGCTCTAAGTGGGTGGTAAATTCCATCTAAAGCTAAATATTGGCGAGAGAC
CGATAGCGAACAAGTACAGTGATGGAAAGATGAAAAGAACTCCGAAAGGAGAG
TGAAAAAGGACGTGAAATTGTTGAAAGGGAAGTGCTTGAGATCAGTCTTAAGGT
TTAGTGATCAGTCTTCCTTTTGGTTGGTGCACTCACTATTTCTTAGGCCAACATCAG
TTTTGGCGGCAGGATAATGACATTGGAATGTGGCTCTTTCGGGAGTGTTATAGCCT
TTGTTGATACTGCCTGCTTAGACTGAGGACTGCGCTTTTGCTAGGATGTTGGCATA
ATGATCTTAAGCAGC

2. ***Lipomyces tetrasporus*** **SWU-NGP 5-3-1**
>SWU-NGP 5-3-1 (561 bp)
AAACCAACAGGGATTGCCTTAGTAACTGCGAGTGAAGCGGCAAGAGCTCAAATT
TGAAATCTGGCACCTTCGGTGTCCGAGTTGTAATTTGTAGAAGCATCTTTTGGATT
GGTCCTTGCATATGTTCCTTGGAACAGGACATCATAGAGGGTGAGAATCCCGTTC
ATGGTGGGGAATCCAATTCTTTGTAAAGTGCTTTCGAAGAGTCGAGTTGTTTGGGA
ATGCAGCTCTAAGTGGGTGGTAAATTCCATCTAAAGCTAAATATTGGCGAGAGAC
CGATAGCGAACAAGTACAGTGATGGAAAGATGAAAAGAACTCCGAAAGGAGAG
TGAAAAAGGACGTGAAATTGTTGAAAGGGAAGTGCTTGAGATCAGTCTTAAGGT
TTAGTGATCAGTCTTCCTTTTGGTTGGTGCACTCACTATTTCTTAGGCCAACATCAG
TTTTCGCGGCAGGATAATGACATTGGAATGTGGCTCTTTCGGGAGTGTTATAGCCT
TTGTTGATACTGCCTGCTTAGACTGAGGACCGCGCTTTTGCTAGGATGTTGGCATA
ATGATCTTA

3. ***Lipomyces tetrasporus*** **SWU-NGP 10-3**

>SWU-NGP 10-3 (567 bp)

AAACCAACAGGGATTGCCTTAGTAACTGCGAGTGAAGCGGCAAGAGCTCAAATT
TGAAATCTGGCACCTTCGGTGTCCGAGTTGTAATTTGTAGAAGCATCTTTTGGATT
GGTCCTTGCATATGTTCCTTGGAACAGGACATCATAGAGGGTGAGAATCCCGTTC
ATGGTGGGGAATCCAATTCTTTGTAAAGTGCTTTCGAAGAGTCGAGTTGTTTGGGA
ATGCAGCTCTAAGTGGGTGGTAAATTCCATCTAAAGCTAAATATTGGCGAGAGAC
CGATAGCGAACAAGTACAGTGATGGAAAGATGAAAAGAACTCCGAAAGGAGAG
TGAAAAAGGACGTGAAATTGTTGAAAGGGAAGTGCTTGAGATCAGTCTTAAGGT
TTAGTGATCAGTCTTCCTTTTGGTTGGTGCACTCACTATTTCTTAGGCCAACATCAG
TTTTCGCGGCAGGATAATGACATTGGAATGTGGCTCTTTCGGGAGTGTTATAGCCT
TTGTTGATACTGCCTGCTTAGACTGAGGACCGCGCTTTTGCTAGGATGTTGGCATA
ATGATCTTAAGCAGC

4. ***Lipomyces tetrasporus*** **SWU-NGP 5-8-1**

>SWU-NGP 5-8-1 (567 bp)

AAACCAACAGGGATTGCCTTAGTAACTGCGAGTGAAGCGGCAAGAGCTCAAATT
TGAAATCTGGCACCTTCGGTGTCCGAGTTGTAATTTGTAGAAGCATCTTTTGGATT
GGTCCTTGCATATGTTCCTTGGAACAGGACATCATAGAGGGTGAGAATCCCGTTC
ATGGTGGGGAATCCAATTCTTTGTAAAGTGCTTTCGAAGAGTCGAGTTGTTTGGGA
ATGCAGCTCTAAGTGGGTGGTAAATTCCATCTAAAGCTAAATATTGGCGAGAGAC
CGATAGCGAACAAGTACAGTGATGGAAAGATGAAAAGAACTCCGAAAGGAGAG
TGAAAAAGGACGTGAAATTGTTGAAAGGGAAGTGCTTGAGATCAGTCTTAAGGT
TTAGTGATCAGTCTTCCTTTTGGTTGGTGCACTCACTATTTCTTAGGCCAACATCAG
TTTTCGCGGCAGGATAATGACATTGGAATGTGGCTCTTTCGGGAGTGTTATAGCCT
TTGTTGATACTGCCTGCTTAGACTGAGGACCGCGCTTTTGCTAGGATGTTGGCATA
ATGATCTTAAGCAGC

5. ***Lipomyces tetrasporus*** **SWU-NGP 11-2**

>SWU-NGP 11-2 (567 bp)

AAACCAACAGGGATTGCCTTAGTAACTGCGAGTGAAGCGGCAAGAGCTCAAATT
TGAAATCTGGCACCTTCGGTGTCCGAGTTGTAATTTGTAGAAGCATCTTTTGGATT
GGTCCTTGCATATGTTCCTTGGAACAGGACATCATAGAGGGTGAGAATCCCGTTC
ATGGTGGGGAATCCAATTCTTTGTAAAGTGCTTTCGAAGAGTCGAGTTGTTTGGGA

ATGCAGCTCTAAGTGGGTGGTAAATTCCATCTAAAGCTAAATATTGGCGAGAGAC
CGATAGCGAACAAGTACAGTGATGGAAAGATGAAAAGAACTCCGAAAGGAGAG
TGAAAAAGGACGTGAAATTGTTGAAAGGGAAGTGCTTGAGATCAGTCTTAAGGT
TTAGTGATCAGTCTTCCTTTTGGTTGGTGCACTCACTATTTCTTAGGCCAACATCAG
TTTTCGCGGCAGGATAATGACATTGGAATGTGGCTCTTTCGGGAGTGTTATAGCCT
TTGTTGATACTGCCTGCTTAGACTGAGGACCGCGCTTTTGCTAGGATGTTGGCATA
ATGATCTTAAGCAGC

6. ***Lipomyces mesembrius*** **SWU-NGP 14-6**
>SWU-NGP 14-6 (567 bp)
AAACCAACAGGGATTGCCTTAGTAACTGCGAGTGAAGCGGCAAGAGCTCAAATT
TGAAATCTGGCACCTTCGGTGTCCGAGTTGTAATTTGTAGAAGCATCTTTTGGATT
GGTCCTTGCATATGTTCCTTGGAACAGGACATCATAGAGGGTGAGAATCCCGTTC
ATGGTGGGGAATCCAATTCTTTGTAAAGTGCTTTCGAAGAGTCGAGTTGTTTGGGA
ATGCAGCTCTAAGTGGGTGGTAAATTCCATCTAAAGCTAAATATTGGCGAGAGAC
CGATAGCGAACAAGTACAGTGATGGAAAGATGAAAAGAACTCCGAAAGGAGAG
TGAAAAAGGACGTGAAATTGTTGAAAGGGAAGTGCTTGAGATCAGTCTTAAGGT
TTAGTGATCAGTCTTCCTTTTGGTTGGTGCACTCACTATTTCTTAGGCCAACATCAG
TTTTGGCGGCAGGATAATGGCATTGGAATGTGGCTCTTTCGGGAGTGTTATAGCCT
TTGTTGATACTGCTTGCTTAGACTGAGGACTGCGCTTTTGCTAGGATGTTGGCATA
ATGATCTTAAGCAGC

7. ***Lipomyces tetrasporu*****s SWU-NGP 1-3**
>SWU-NGP 1-3 (567 bp)
AAACCAACAGGGATTGCCTTAGTAACTGCGAGTGAAGCGGCAAGAGCTCAAATT
TGAAATCTGGCACCTTCGGTGTCCGAGTTGTAATTTGTAGAAGCATCTTTTGGATT
GGTCCTTGCATATGTTCCTTGGAACAGGACATCATAGAGGGTGAGAATCCCGTTC
ATGGTGGGGAATCCAATTCTTTGTAAAGTGCTTTCGAAGAGTCGAGTTGTTTGGGA
ATGCAGCTCTAAGTGGGTGGTAAATTCCATCTAAAGCTAAATATTGGCGAGAGAC
CGATAGCGAACAAGTACAGTGATGGAAAGATGAAAAGAACTCCGAAAGGAGAG
TGAAAAAGGACGTGAAATTGTTGAAAGGGAAGTGCTTGAGATCAGTCTTAAGGT
TTAGTGATCAGTCTTCCTTTTGGTTGGTGCACTCACTATTTCTTAGGCCAACATCAG
TTTTCGCGGCAGGATAATGACATTGGAATGTGGCTCTTTCGGGAGTGTTATAGCCT

TTGTTGATACTGCCTGCTTAGACTGAGGACCGCGCTTTTGCTAGGATGTTGGCATA
ATGATCTTAAGCAGC

8. *Lipomyces tetrasporus* SWU-NGP 9-3
>SWU-NGP 9-3 (567 bp)
AAACCAACAGGGATTGCCTTAGTAACTGCGAGTGAAGCGGCAAGAGCTCAAATT
TGAAATCTGGCACCTTCGGTGTCCGAGTTGTAATTTGTAGAAGCATCTTTTGGATT
GGTCCTTGCATATGTTCCTTGGAACAGGACATCATAGAGGGTGAGAATCCCGTTC
ATGGTGGGGAATCCAATTCTTTGTAAAGTGCTTTCGAAGAGTCGAGTTGTTTGGGA
ATGCAGCTCTAAGTGGGTGGTAAATTCCATCTAAAGCTAAATATTGGCGAGAGAC
CGATAGCGAACAAGTACAGTGATGGAAAGATGAAAAGAACTCCGAAAGGAGAG
TGAAAAAGGACGTGAAATTGTTGAAAGGGAAGTGCTTGAGATCAGTCTTAAGGT
TTAGTGATCAGTCTTCCTTTTGGTTGGTGCACTCACTATTTCTTAGGCCAACATCAG
TTTTCGCGGCAGGATAATGACATTGGAATGTGGCTCTTTCGGGAGTGTTATAGCCT
TTGTTGATACTGCCTGCTTAGACTGAGGACCGCGCTTTTGCTAGGATGTTGGCATA
ATGATCTTAAGCAGC

9. *Lipomyces starkeyi* SWU-NGTP 5-7
> SWU-NGTP 5-7 (526 bp)
AAGAGCTCAAATTTGAAATCTGGCACCTTCGGTGTCCGAGTTGTAATTTGTAGAA
GCATCTTTTGGATTGGTCCTTGCATATGTTCCTTGGAACAGGACATCATAGAGGGT
GAGAATCCCGTTCATGGTGGGGAATCCAATTCTTTGTAAAGTGCTTTCGAAGAGT
CGAGTTGTTTGGGAATGCAGCTCTAAGTGGGTGGTAAATTCCATCTAAAGCTAAA
TATTGGCGAGAGACCGATAGCGAACAAGTACAGTGATGGAAAGATGAAAAGAA
CTCCGAAAGGAGAGTGAAAAAGGACGTGAAATTGTTGAAAGGGAAGTGCTTGAG
ATCAGTCTTAAGGTTTAGTGATCAGTCTTCCTTTTGGTTGGTGCACTCACTATTTCT
TAGGCCAACATCAGTTTTGGCGGCAGGATAATGACATTGGAATGTGGCTCTTTCG
GGAGTGTTATAGCCTTTGTTGATACTGCCTGCTTAGACTGAGGACTGCGCTTTTGC
TAGGATGTTGGCATAATGATCTTAAGCAGC

10. ***Lipomyces tetrasporus*** **SWU-NGP 11-1**

>SWU-NGP 11-1 (567 bp)

AAACCAACAGGGATTGCCTTAGTAACTGCGAGTGAAGCGGCAAGAGCTCAAATT
TGAAATCTGGCACCTTCGGTGTCCGAGTTGTAATTTGTAGAAGCATCTTTTGGATT
GGTCCTTGCATATGTTCCTTGGAACAGGACATCATAGAGGGTGAGAATCCCGTTC
ATGGTGGGGAATCCAATTCTTTGTAAAGTGCTTTCGAAGAGTCGAGTTGTTTGGGA
ATGCAGCTCTAAGTGGGTGGTAAATTCCATCTAAAGCTAAATATTGGCGAGAGAC
CGATAGCGAACAAGTACAGTGATGGAAAGATGAAAAGAACTCCGAAAGGAGAG
TGAAAAAGGACGTGAAATTGTTGAAAGGGAAGTGCTTGAGATCAGTCTTAAGGT
TTAGTGATCAGTCTTCCTTTTGGTTGGTGCACTCACTATTTCTTAGGCCAACATCAG
TTTTCGCGGCAGGATAATGACATTGGAATGTGGCTCTTTCGGGAGTGTTATAGCCT
TTGTTGATACTGCCTGCTTAGACTGAGGACCGCGCTTTTGCTAGGATGTTGGCATA
ATGATCTTAAGCAGC

11. ***Lipomyces tetrasporus*** **SWU-NGP 4-2-2**

>SWU-NGP 4-2-2 (560 bp)

AAACCAACAGGGATTGCCTTAGTAACTGCGAGTGAAGCGGCAAGAGCTCAAATT
TGAAATCTGGCACCTTCGGTGTCCGAGTTGTAATTTGTAGAAGCATCTTTTGGATT
GGTCCTTGCATATGTTCCTTGGAACAGGACATCATAGAGGGTGAGAATCCCGTTC
ATGGTGGGGAATCCAATTCTTTGTAAAGTGCTTTCGAAGAGTCGAGTTGTTTGGGA
ATGCAGCTCTAAGTGGGTGGTAAATTCCATCTAAAGCTAAATATTGGCGAGAGAC
CGATAGCGAACAAGTACAGTGATGGAAAGATGAAAAGAACTCCGAAAGGAGAG
TGAAAAAGGACGTGAAATTGTTGAAAGGGAAGTGCTTGAGATCAGTCTTAAGGT
TTAGTGATCAGTCTTCCTTTTGGTTGGTGCACTCACTATTTCTTAGGCCAACATCAG
TTTTCGCGGCAGGATAATGACATTGGAATGTGGCTCTTTCGGGAGTGTTATAGCCT
TTGTTGATACTGCCTGCTTAGACTGAGGACCGCGCTTTTGCTAGGATGTTGGCATA
ATGATCTT

12. ***Lipomyces tetrasporus*** **SWU-NGP 4-14**

>SWU-NGP 4-14 (486 bp)

AAACCAACAGGGATTGCCTTAGTAACTGCGAGTGAAGCGGCAAGAGCTCAAATT
TGAAATCTGGCACCTTCGGTGTCCGAGTTGTAATTTGTAGAAGCATCTTTTGGATT
GGTCCTTGCATATGTTCCTTGGAACAGGACATCATAGAGGGTGAGAATCCCGTTC
ATGGTGGGGAATCCAATTCTTTGTAAAGTGCTTTCGAAGAGTCGAGTTGTTTGGGA

ATGCAGCTCTAAGTGGGTGGTAAATTCCATCTAAAGCTAAATATTGGCGAGAGAC
CGATAGCGAACAAGTACAGTGATGGAAAGATGAAAAGAACTCCGAAAGGAGAG
TGAAAAAGGACGTGAAATTGTTGAAAGGGAAGTGCTTGAGATCAGTCTTAAGGT
TTAGTGATCAGTCTTCCTTTTGGTTGGTGCACTCACTATTTCTTAGGCCAACATCAG
TTTTCGCGGCAGGATAATGACATTGGAATGTGGCTCTTTCGGGAGT

13. ***Lipomyces starkeyi*** **SWU-NGTP 5-7-1**

>SWU-NGTP 5-7-1 (485 bp)

TGTCCGAGTTGTAATTTGTAGAAGCATCTTTTGGATTGGTCCTTGCATATGTTCCTT
GGAACAGGACATCATAGAGGGTGAGAATCCCGTTCATGGTGGGGAATCCAATTC
TTTGTAAAGTGCTTTCGAAGAGTCGAGTTGTTTGGGAATGCAGCTCTAAGTGGGTG
GTAAATTCCATCTAAAGCTAAATATTGGCGAGAGACCGATAGCGAACAAGTACA
GTGATGGAAAGATGAAAAGAACTCCGAAAGGAGAGTGAAAAAGGACGTGAAAT
TGTTGAAAGGGAAGTGCTTGAGATCAGTCTTAAGGTTTAGTGATCAGTCTTCCTTT
TGGTTGGTGCACTCACTATTTCTTAGGCCAACATCAGTTTTGGCGGCAGGATAATG
ACATTGGAATGTGGCTCTTTCGGGAGTGTTATAGCCTTTGTTGATACTGCCTGCTT
AGACTGAGGACTGCGCTTTTGCTAGGATGTTGGCATAATGATCTTAAGCAGC

14. ***Lipomyces tetrasporu*****s SWU-NAP 2-1**

>SWU-NAP 2-1 (567 bp)

AAACCAACAGGGATTGCCTTAGTAACTGCGAGTGAAGCGGCAAGAGCTCAAATT
TGAAATCTGGCACCTTCGGTGTCCGAGTTGTAATTTGTAGAAGCATCTTTTGGATT
GGTCCTTGCATATGTTCCTTGGAACAGGACATCATAGAGGGTGAGAATCCCGTTC
ATGGTGGGGAATCCAATTCTTTGTAAAGTGCTTTCGAAGAGTCGAGTTGTTTGGGA
ATGCAGCTCTAAGTGGGTGGTAAATTCCATCTAAAGCTAAATATTGGCGAGAGAC
CGATAGCGAACAAGTACAGTGATGGAAAGATGAAAAGAACTCCGAAAGGAGAG
TGAAAAAGGACGTGAAATTGTTGAAAGGGAAGTGCTTGAGATCAGTCTTAAGGT
TTAGTGATCAGTCTTCCTTTTGGTTGGTGCACTCACTATTTCTTAGGCCAACATCAG
TTTTCGCGGCAGGATAATGACATTGGAATGTGGCTCTTTCGGGAGTGTTATAGCCT
TTGTTGATACTGCCTGCTTAGACTGAGGACCGCGCTTTTGCTAGGATGTTGGCATA
ATGATCTTAAGCAGC

15. ***Lipomyces mesembrius*** **SWU-NGP 14-8**

>SWU-NGP 14-8 (567 bp)

AAACCAACAGGGATTGCCTTAGTAACTGCGAGTGAAGCGGCAAGAGCTCAAATT
TGAAATCTGGCACCTTCGGTGTCCGAGTTGTAATTTGTAGAAGCATCTTTTGGATT
GGTCCTTGCATATGTTCCTTGGAACAGGACATCATAGAGGGTGAGAATCCCGTTC
ATGGTGGGGAATCCAATTCTTTGTAAAGTGCTTTCGAAGAGTCGAGTTGTTTGGGA
ATGCAGCTCTAAGTGGGTGGTAAATTCCATCTAAAGCTAAATATTGGCGAGAGAC
CGATAGCGAACAAGTACAGTGATGGAAAGATGAAAAGAACTCCGAAAGGAGAG
TGAAAAAGGACGTGAAATTGTTGAAAGGGAAGTGCTTGAGATCAGTCTTAAGGT
TTAGTGATCAGTCTTCCTTTTGGTTGGTGCACTCACTATTTCTTAGGCCAACATCAG
TTTTGGCGGCAGGATAATGGCATTGGAATGTGGCTCTTTCGGGAGTGTTATAGCCT
TTGTTGATACTGCTTGCTTAGACTGAGGACTGCGCTTTTGCTAGGATGTTGGCATA
ATGATCTTAAGCAGC

16. ***Lipomyces mesembrius*** **SWU-NGP 6-4**

>SWU-NGP 6-4 (567 bp)

AAACCAACAGGGATTGCCTTAGTAACTGCGAGTGAAGCGGCAAGAGCTCAAATT
TGAAATCTGGCACCTTCGGTGTCCGAGTTGTAATTTGTAGAAGCATCTTTTGGATT
GGTCCTTGCATATGTTCCTTGGAACAGGACATCATAGAGGGTGAGAATCCCGTTC
ATGGTGGGGAATCCAATTCTTTGTAAAGTGCTTTCGAAGAGTCGAGTTGTTTGGGA
ATGCAGCTCTAAGTGGGTGGTAAATTCCATCTAAAGCTAAATATTGGCGAGAGAC
CGATAGCGAACAAGTACAGTGATGGAAAGATGAAAAGAACTCCGAAAGGAGAG
TGAAAAAGGACGTGAAATTGTTGAAAGGGAAGTGCTTGAGATCAGTCTTAAGGT
TTAGTGATCAGTCTTCCTTTTGGTTGGTGCACTCACTATTTCTTAGGCCAACATCAG
TTTTGGCGGCAGGATAATGACATTGGAATGTGGCTCTTTCGGGAGTGTTATAGCCT
TTGTTGATACTGCCTGCTTAGACTGAGGACTGCGCTTTTGCTAGGATGTTGGCATA
ATGATCTTAAGCAGC

17. ***Lipomyces tetrasporu*s** **SWU-NAP 9-2**

>SWU-NAP 9-2 (567 bp)

AAACCAACAGGGATTGCCTTAGTAACTGCGAGTGAAGCGGCAAGAGCTCAAATT
TGAAATCTGGCACCTTCGGTGTCCGAGTTGTAATTTGTAGAAGCATCTTTTGGATT
GGTCCTTGCATATGTTCCTTGGAACAGGACATCATAGAGGGTGAGAATCCCGTTC
ATGGTGGGGAATCCAATTCTTTGTAAAGTGCTTTCGAAGAGTCGAGTTGTTTGGGA

ATGCAGCTCTAAGTGGGTGGTAAATTCCATCTAAAGCTAAATATTGGCGAGAGAC
CGATAGCGAACAAGTACAGTGATGGAAAGATGAAAAGAACTCCGAAAGGAGAG
TGAAAAAGGACGTGAAATTGTTGAAAGGGAAGTGCTTGAGATCAGTCTTAAGGT
TTAGTGATCAGTCTTCCTTTTGGTTGGTGCACTCACTATTTCTTAGGCCAACATCAG
TTTTCGCGGCAGGATAATGACATTGGAATGTGGCTCTTTCGGGAGTGTTATAGCCT
TTGTTGATACTGCCTGCTTAGACTGAGGACCGCGCTTTTGCTAGGATGTTGGCATA
ATGATCTTAAGCAGC

18. ***Lipomyces tetrasporus*** **SWU-NAP 5-4**
>SWU-NAP 5-4 (567 bp)
AAACCAACAGGGATTGCCTTAGTAACTGCGAGTGAAGCGGCAAGAGCTCAAATT
TGAAATCTGGCACCTTCGGTGTCCGAGTTGTAATTTGTAGAAGCATCTTTTGGATT
GGTCCTTGCATATGTTCCTTGGAACAGGACATCATAGAGGGTGAGAATCCCGTTC
ATGGTGGGGAATCCAATTCTTTGTAAAGTGCTTTCGAAGAGTCGAGTTGTTTGGGA
ATGCAGCTCTAAGTGGGTGGTAAATTCCATCTAAAGCTAAATATTGGCGAGAGAC
CGATAGCGAACAAGTACAGTGATGGAAAGATGAAAAGAACTCCGAAAGGAGAG
TGAAAAAGGACGTGAAATTGTTGAAAGGGAAGTGCTTGAGATCAGTCTTAAGGT
TTAGTGATCAGTCTTCCTTTTGGTTGGTGCACTCACTATTTCTTAGGCCAACATCAG
TTTTCGCGGCAGGATAATGACATTGGAATGTGGCTCTTTCGGGAGTGTTATAGCCT
TTGTTGATACTGCCTGCTTAGACTGAGGACCGCGCTTTTGCTAGGATGTTGGCATA
ATGATCTTAAGCAGC

19. ***Lipomyces tetrasporus*** **SWU-NGP 3-7**
>SWU-NGP 3-7 (508 bp)
AAACCAACAGGGATTGCCTTAGTAACTGCGAGTGAAGCGGCAAGAGCTCAAATT
TGAAATCTGGCACCTTCGGTGTCCGAGTTGTAATTTGTAGAAGCATCTTTTGGATT
GGTCCTTGCATATGTTCCTTGGAACAGGACATCATAGAGGGTGAGAATCCCGTTC
ATGGTGGGGAATCCAATTCTTTGTAAAGTGCTTTCGAAGAGTCGAGTTGTTTGGGA
ATGCAGCTCTAAGTGGGTGGTAAATTCCATCTAAAGCTAAATATTGGCGAGAGAC
CGATAGCGAACAAGTACAGTGATGGAAAGATGAAAAGAACTCCGAAAGGAGAG
TGAAAAAGGACGTGAAATTGTTGAAAGGGAAGTGCTTGAGATCAGTCTTAAGGT
TTAGTGATCAGTCTTCCTTTTGGTTGGTGCACTCACTATTTCTTAGGCCAACATCAG
TTTTCGCGGCAGGATAATGACATTGGAATGTGGCTCTTTCGGGAGTGTTATAGCCT
TTGTTGATACTG

20. ***Lipomyces starkeyi*** **SWU- NAP 13-4**

>SWU-NAP 13-4 (567 bp)

AAACCAACAGGGATTGCCTTAGTAACTGCGAGTGAAGCGGCAAGAGCTCAAATT
TGAAATCTGGCACCTTCGGTGTCCGAGTTGTAATTTGTAGAAGCATCTTTTGGATT
GGTCCTTGCATATGTTCCTTGGAACAGGACATCATAGAGGGTGAGAATCCCGTTC
ATGGTGGGGAATCCAATTCTTTGTAAAGTGCTTTCGAAGAGTCGAGTTGTTTGGGA
ATGCAGCTCTAAGTGGGTGGTAAATTCCATCTAAAGCTAAATATTGGCGAGAGAC
CGATAGCGAACAAGTACAGTGATGGAAAGATGAAAAGAACTCCGAAAGGAGAG
TGAAAAAGGACGTGAAATTGTTGAAAGGGAAGTGCTTGAGATCAGTCTTAAGGT
TTAGTGATCAGTCTTCCTTTTGGTTGGTGCACTCACTATTTCTTAGGCCAACATCAG
TTTTGGCGGCAGGATAATGACATTGGAATGTGGCTCTTTCGGGAGTGTTATAGCCT
TTGTTGATACTGCCTGCTTAGACTGAGGACTGCGCTTTTGCTAGGATGTTGGCATA
ATGATCTTAAGCAGC

21. ***Papiliotrema terrestris*** **SWU-NAPui 14-5**

>SWU-NAPui 14-5 (596 bp)

AAACTAACAAGGATTCCCCTAGTAACGGCGAGTGAACCGGGAAGAGCTCAAATT
TGAAATCTGGCGTGCTCAGTGCGTCCGAGTTGTAATCTATAGAGGCGTTTTCCGTG
CCGGACTGTGTCTAAGTCCCTTGGAACAGGGTATCAAAGAGGGTGATAATCCCGT
ACTTGACACAATGACCGGTGCTCTGTGATACGTCTTCTACGAGTCGAGTTGTTTGG
GAATGCAGCTCAAAATGGGTGGTGAGTTCCATCTAAAGCTAAATATTGGCGAGA
GACCGATAGCGAACAAGTACCGTGAGGGAAAGATGAAAAGCACTTTGGAAAGA
GAGTTAAACAGTACGTGAAATTGTTGAAAGGGAAACGATTGAAGTCAGTCGTGA
CTGAGAGGCTCAGCCGGTTCTGCCGGTGTATTCCCCTCAGTCGGGTCAACATCAG
TTTTGTTCGGTGGATAAGGGCAGCTGGAAGGTGGCACCTCCGGGTGTGTTATAGC
CAGCTGTCGCATACATCGAATGAGACTGAGGAATGCAGCTCGCCTTTATGGCCGG
GGTTCGCCCACGTCCGAGCTTAGGATGTTGACATAATGGCTTTAAACGA

22. ***Cyberlindnera saturnus*** **SWU-NATP 4-4**

>SWU-NATP 4-4 (574 bp)

AAACCAACAGGGATTGCCTCAGTAACGGCGAGTGAAGCGGCAAAAGCTCAAATT
TGAAATCTGGTACCTTTGGTGCCCGAGTTGTAATTTGAAGATAGTTTTCTGGTGCT
GGCCCTTGTCTATGTTCCTTGGAACAGGACGTCACAGAGGGTGAGAATCCCGTCT

GGCGGGGTGTCCAGTGCTTTGTAGATTTCTATCGACGAGTCGAGTTGTTTGGGAAT
GCAGCTCTAAGTGGGTGGTAAATTCCATCTAAAGCTAAATATTGGCGAGAGACCG
ATAGCGAACAAGTACAGTGATGGAAAGATGAAAAGAACTTTGAAAAGAGAGTG
AAAAAGTACGTGAAATTGTTGAAAGGGAAGGGTATTAGATCAGACTTGGTGTTTT
GTGATTATCTTCCCTTCTTGGGTTGTGCACTCGCATTTCACTGGGCCAGCATCGGTT
CGGGTGGTAAGATAATGACATTGGAACGTGGCACTACCTTCGGGTGGTGTGTTAT
AGCCCTTGTTGATGTTGCCTACCTGGACCGAGGACTGCGGCTTTTGCCTAGGATGC
TGGCGTAATGATCTAACACCGC

23. ***Piskurozyma* sp. SWU-NATP 4-12**
>SWU-NATP 4-12 (596 pb)
TAACAAGGATTCCCCTAGTAACGGCGAGTGAAGCGGGAAGAGCTCAAATTTGAA
ATCTGGCGCCCTCAGGACGTCCGAGTTGTAATCTATAGGAACGTTTTCCGTGCCG
GCTCATGTACAAGTCCCTTGGAACAGGGCGTCATAGAGGGTGAGAATCCCGTACT
TGACATGAACCCCCGGTGCTCTGTGATACGTCTCCAACGAGTCGAGTTGTTTGGG
AATGCAGCTCAAAATGGGTGGTAAATTCCATCTAAAGCTAAATATTGGTGTGAGA
CCGATAGCGAACAAGTACCGTGAGGGAAAGATGAAAAGCACTTTGGAAAGAGA
GTTAAAAAGTATGTGAAATTGTTGAAAGGGAAACGATTGAAGTCAGTCGTGTCCT
TCAGATTCAGCTGGTTCTGCCGGTGTATTTCTGTTGGATGGGGTCAACATCAGTTT
CGGATGCCGGATAAGGGCTGGAGGAAGGTAGTACCCCCGGGTAAACTTATAGCC
TCCTGTCGCATACGGTGACTGGGACTGAGGAACGCAGCTTGCCTTCATGGCCGGG
GTTCGCCCACGTACAAGCTTAGGATGTTGACATAATGGCTTTAAACGAC

24. ***Papiliotrema flavescens* SWU-NATP 2-7**
>SWU-NATP 2-7 (587 bp)
TAACAAGGATTCCCCTAGTAACGGCGAGTGAACCGGGAAGAGCTCAAATTTGAA
ATCTGGCGTGCTCAGTGCGTCCGAGTTGTAATCTATAGAGTCGTTTTCCGTGCCGG
ACTGTGTCCAAGTCCCTTGGAACAGGGTATCAAAGAGGGTGATAATCCCGTACTT
GACACAATGACCGGTGCTCTGTGATACGTCTTCTACGAGTCGAGTTGTTTGGGAA
TGCAGCTCAAAATGGGTGGTGAGTTCCATCTAAAGCTAAATATTGGCGAGAGACC
GATAGCGAACAAGTACCGTGAGGGAAAGATGAAAAGCACTTTGGAAAGAGAGT
TAAACAGTACGTGAAATTGTTAAAAGGGAAACGATTGAAGTCAGTCGTGACTGA
GAGGCTCAGCCGGTTCTGCCGGTGTATTCCCCTCAGTCGGGTCAACATCAGTTTTG

TTCGGTGGATAAGGGCAGTTGGAAGGTGGCACCCTCGGGTGTGTTATAGCCAGCT
GTCGCATACATCGGATGAGACTGAGGAATGCAGCTCGCCTTTATGGCCGGGGTTC
GCCCACGTTCGAGCTTAGGATGTTGACATAATGGCTTTA

25. ***Lipomyces tetrasporus*** **NAP 13-8**
>SWU-NAP 13-8 (567 bp)
AAACCAACAGGGATTGCCTTAGTAACTGCGAGTGAAGCGGCAAGAGCTCAAATT
TGAAATCTGGCACCTTCGGTGTCCGAGTTGTAATTTGTAGAAGCATCTTTTGGATT
GGTCCTTGCATATGTTCCTTGGAACAGGACATCATAGAGGGTGAGAATCCCGTTC
ATGGTGGGGAATCCAATTCTTTGTAAAGTGCTTTCGAAGAGTCGAGTTGTTTGGGA
ATGCAGCTCTAAGTGGGTGGTAAATTCCATCTAAAGCTAAATATTGGCGAGAGAC
CGATAGCGAACAAGTACAGTGATGGAAAGATGAAAAGAACTCCGAAAGGAGAG
TGAAAAAGGACGTGAAATTGTTGAAAGGGAAGTGCTTGAGATCAGTCTTAAGGT
TTAGTGATCAGTCTTCCTTTTGGTTGGTGCACTCACTATTTCTTAGGCCAACATCAG
TTTTCGCGGCAGGATAATGACATTGGAATGTGGCTCTTTCGGGAGTGTTATAGCCT
TTGTTGATACTGCCTGCTTAGACTGAGGACCGCGCTTTTGCTAGGATGTTGGCATA
ATGATCTTAAGCAGC

26. ***Lipomyces mesembrius*** **SWU-NAP 3-4**
>SWU-NAP 3-4 (567 bp)
AAACCAACAGGGATTGCCTTAGTAACTGCGAGTGAAGCGGCAAGAGCTCAAATT
TGAAATCTGGCACCTTCGGTGTCCGAGTTGTAATTTGTAGAAGCATCTTTTGGATT
GGTCCTTGCATATGTTCCTTGGAACAGGACATCATAGAGGGTGAGAATCCCGTTC
ATGGTGGGGAATCCAATTCTTTGTAAAGTGCTTTCGAAGAGTCGAGTTGTTTGGGA
ATGCAGCTCTAAGTGGGTGGTAAATTCCATCTAAAGCTAAATATTGGCGAGAGAC
CGATAGCGAACAAGTACAGTGATGGAAAGATGAAAAGAACTCCGAAAGGAGAG
TGAAAAAGGACGTGAAATTGTTGAAAGGGAAGTGCTTGAGATCAGTCTTAAGGT
TTAGTGATCAGTCTTCCTTTTGGTTGGTGCACTCACTATTTCTTAGGCCAACATCAG
TTTTGGCGGCAGGATAATGACATTGGAATGTGGCTCTTTCGGGAGTGTTATAGCCT
TTGTTGATACTGCCTGCTTAGACTGAGGACTGCGCTTTTGCTAGGATGTTGGCATA
ATGATCTTAAGCAG

27. ***Lipomyces mesembrius*** **SWU-NAP 8-2**

>SWU-NAP 8-2 (567 bp)

AAACCAACAGGGATTGCCTTAGTAACTGCGAGTGAAGCGGCAAGAGCTCAAATT
TGAAATCTGGCACCTTCGGTGTCCGAGTTGTAATTTGTAGAAGCATCTTTTGGATT
GGTCCTTGCATATGTTCCTTGGAACAGGACATCATAGAGGGTGAGAATCCCGTTC
ATGGTGGGGAATCCAATTCTTTGTAAAGTGCTTTCGAAGAGTCGAGTTGTTTGGGA
ATGCAGCTCTAAGTGGGTGGTAAATTCCATCTAAAGCTAAATATTGGCGAGAGAC
CGATAGCGAACAAGTACAGTGATGGAAAGATGAAAAGAACTCCGAAAGGAGAG
TGAAAAAGGACGTGAAATTGTTGAAAGGGAAGTGCTTGAGATCAGTCTTAAGGT
TTAGTGATCAGTCTTCCTTTTGGTTGGTGCACTCACTATTTCTTAGGCCAACATCAG
TTTTGGCGGCAGGATAATGACATTGGAATGTGGCTCTTTCGGGAGTGTTATAGCCT
TTGTTGATACTGCCTGCTTAGACTGAGGACTGCGCTTTTGCTAGGATGTTGGCATA
ATGATCTTAAGCAGC

28. ***Saitozyma podzolica*** **SWU-YGP 8-1-2**

>SWU-YGP 8-1-2 (597 bp)

AAACTAACAAGGATTCCCTTAGTAGCGGCGAGCGAAGCGGGAAGAGCTCAAATT
TGAAAGCTGGCGTCCTACGGGCGTCCGCATTGTAATCTATAGAGGCGTTTTCTGTG
CTGGACCGTGTCCAAGTCCCTTGGAACAGGGTATCAAAGAGGGTGACAATCCCG
TACTTGACACGACAACCAGTGCTCTGTGATACGTCTTCTACGAGTCGAGTTGTTTG
GGAATGCAGCTCAAAATGGGTGGTGAATTCCATCTAAAGCTAAATATTGGCGAG
AGACCGATAGCGAACAAGTACCGTGAGGGAAAGATGAAAAGCACTTTGGAAAG
AGAGTTAAACAGTATGTGAAATTGTTGAAAGGGAAACGATGGAGGTCAGTCGTG
TCTGTGGGATTCAGCCGTCTCTGGCGGTGTACTTCCCACAGACGGGTCAACATCA
GTTTTGGCTGGCGGATAAAGGCAGGAGGAAGGTGGCACCCCCGGGTGTGTTATA
GCCTCTTGTTGCATACGCTGGCTGAGACTGAGGAATGCAGCTCGCCTTTATGGCC
GGGGTTCGCCCACGTTCGAGCTTAGGATGTTGACATAATGGCCTTCAACGAC

29. ***Cyberlindnera saturnus*** **SWU-NATP 4-6**

>SWU-NATP 4-6 (580 bp)

AAACCAACAGGGATTGCCTCAGTAACGGCGAGTGAAGCGGCAAAAGCTCAAATT
TGAAATCTGGTACCTTTGGTGCCCGAGTTGTAATTTGAAGATAGTTTTCTGGTGCT
GGCCCTTGTCTATGTTCCTTGGAACAGGACGTCACAGAGGGTGAGAATCCCGTCT
GGCGGGGTGTCCAGTGCTTTGTAGATTTCTATCGACGAGTCGAGTTGTTTGGGAAT

GCAGCTCTAAGTGGGTGGTAAATTCCATCTAAAGCTAAATATTGGCGAGAGACCG
ATAGCGAACAAGTACAGTGATGGAAAGATGAAAAGAACTTTGAAAAGAGAGTG
AAAAAGTACGTGAAATTGTTGAAAGGGAAGGGTATTAGATCAGACTTGGTGTTTT
GTGATTATCTTCCCTTCTTGGGTTGTGCACTCGCATTTCACTGGGCCAGCATCGGTT
CGGGTGGTAAGATAATGACATTGGAACGTGGCACTACCTTCGGGTGGTGTGTTAT
AGCCCTTGTTGATGTTGCCTACCTGGACCGAGGACTGCGGCTTTTGCCTAGGATGC
TGGCGTAATGATCTAACACCGC

30. ***Cyberlindnera saturnus*** **SWU-NGTP 4-14-3**

>SWU-NGTP 4-14-3 (574 bp)

AAACCAACAGGGATTGCCTCAGTAACGGCGAGTGAAGCGGCAAAAGCTCAAATT
TGAAATCTGGTACCTTTGGTGCCCGAGTTGTAATTTGAAGATAGTTTTCTGGTGCT
GGCCCTTGTCTATGTTCCTTGGAACAGGACGTCACAGAGGGTGAGAATCCCGTCT
GGCGGGGTGTCCAGTGCTTTGTAGATTTCTATCGACGAGTCGAGTTGTTTGGGAAT
GCAGCTCTAAGTGGGTGGTAAATTCCATCTAAAGCTAAATATTGGCGAGAGACCG
ATAGCGAACAAGTACAGTGATGGAAAGATGAAAAGAACTTTGAAAAGAGAGTG
AAAAAGTACGTGAAATTGTTGAAAGGGAAGGGTATTAGATCAGACTTGGTGTTTT
GTGATTATCTTCCCTTCTTGGGTTGTGCACTCGCATTTCACTGGGCCAGCATCGGTT
CGGGTGGTAAGATAATGACATTGGAACGTGGCACTACCTTCGGGTGGTGTGTTAT
AGCCCTTGTTGATGTTGCCTACCTGGACCGAGGACTGCGGCTTTTGCCTAGGATGC
TGGCGTAATGATCTAACACCGC

31. ***Papiliotrema flavescen*****s SWU-NGTP 3-2**

>SWU-NGTP 3-2 (584 bp)

GATTCCCCTAGTAACGGCGAGTGAACCGGGAAGAGCTCAAATTTGAAATCTGGC
GTGCTCAGTGCGTCCGAGTTGTAATCTATAGAGTCGTTTTCCGTGCCGGACTGTGT
CCAAGTCCCTTGGAACAGGGTATCAAAGAGGGTGATAATCCCGTACTTGACACA
ATGACCGGTGCTCTGTGATACGTCTTCTACGAGTCGAGTTGTTTGGGAATGCAGCT
CAAAATGGGTGGTGAGTTCCATCTAAAGCTAAATATTGGCGAGAGACCGATAGC
GAACAAGTACCGTGAGGGAAAGATGAAAAGCACTTTGGAAAGAGAGTTAAACA
GTACGTGAAATTGTTAAAAGGGAAACGATTGAAGTCAGTCGTGACTGAGAGGCT
CAGCCGGTTCTGCCGGTGTATTCCCCTCAGTCGGGTCAACATCAGTTTTGTTCGGT
GGATAAGGGCAGTTGGAAGGTGGCACCCTCGGGTGTGTTATAGCCAGCTGTCGCA

TACATCGGATGAGACTGAGGAATGCAGCTCGCCTTTATGGCCGGGGTTCGCCCAC
GTTCGAGCTTAGGATGTTGACATAATGGCTTTAAACG

32. ***Papiliotrema terrestris*** **SWU-NAPui 12-9**
>SWU-NAPui 12-9 (598 bp)
AAACTAACAAGGATTCCCCTAGTAACGGCGAGTGAACCGGGAAGAGCTCAAATT
TGAAATCTGGCGTGCTCAGTGCGTCCGAGTTGTAATCTATAGAGGCGTTTTCCGTG
CCGGACTGTGTCTAAGTCCCTTGGAACAGGGTATCAAAGAGGGTGATAATCCCGT
ACTTGACACAATGACCGGTGCTCTGTGATACGTCTTCTACGAGTCGAGTTGTTTGG
GAATGCAGCTCAAAATGGGTGGTGAGTTCCATCTAAAGCTAAATATTGGCGAGA
GACCGATAGCGAACAAGTACCGTGAGGGAAAGATGAAAAGCACTTTGGAAAGA
GAGTTAAACAGTACGTGAAATTGTTGAAAGGGAAACGATTGAAGTCAGTCGTGA
CTGAGAGGCTCAGCCGGTTCTGCCGGTGTATTCCCCTCAGTCGGGTCAACATCAG
TTTTGTTCGGTGGATAAGGGCAGCTGGAAGGTGGCACCTCCGGGTGTGTTATAGC
CAGCTGTCGCATACATCGAATGAGACTGAGGAATGCAGCTCGCCTTTATGGCCGG
GGTTCGCCCACGTCCGAGCTTAGGATGTTGACATAATGGCTTTAAACGACC

33. ***Naganishia diffluens*** **SWU-NGP 1-5-1**
>SWU-NGP 1-5-1 (576 bp)
AACAAGGATTCCCCTAGTAACGGCGAGTGAAGCGGGAAGAGCTCAAATTTGAAA
TCTAGTAGCCTTCGGCTGCTCGAGTTGTAATCTAGAGAAGTGTTTTCCGTGCCGGC
CCATGTACAAGTCCCTTGGAACAGGGCGTCATAGAGGGTGAGAATCCCGTCCTTG
ACATGGACCCCCGGTGCTCTGTGATACACTTTCAACGAGTCGAGTTGTTTGGGAA
TGCAGCTCAAAATGGGTGGTGAATTCCATCTAAAGCTAAATATTGGCGAGAGACC
GATAGCGAACAAGTACCGTGAGGGAAAGATGAAAAGCACTTTGGAAAGAGAGT
TAAACAGTACGTGAAATTGTTGAAAGGGAAACGATTGAAGTCAGTCATGCTCTTT
GGTATTTATATCATTGAGTGGGGTCAACATCAGTTTTGAGCGATGGATAAAGGCA
CTAGGAAGGTAGCACTCTCGGGTGAACTTATAGCCCAGCGTCATATACATTGTTT
GGGACTGAGGAACGCAGCATGCCTTTATGGCCGGGATTCGTCCACGTACATGCTT
AGGATGTTGACATAATGGCTTTAAACGA

34. ***Cystobasidium slooffiae*** **SWU-NAP 4-2-2**

>SWU-NAP 4-2-2 (583 bp)

CCCTAGTAACGGCGAGTGAAGTGGAAAAGCTCAACTTTGAAATCTGGCACCTTC
GGTGTCCGAGTTGTAGTCTCAAGAAGTGTTTTCTGTGCTGGTCCATGTATGAGTCT
GTTGGAACACAGCGTCATAGAGGGTGACAACCCCGTTCATGACATGGATACCAG
TGCTTTGTGATACACTCTCGAAGAGTCGAGTTGTTTGGGAATGCAGCTCAAATTGG
GTGGTAAATTCCATCTAAAGCTAAATATTGGCGAGAGACCGATAGCAAACAAGT
ACCGTGAGGGAAAGATGAAAAGCACTTTGGAAAGAGAGTTAACAGTACGTGAA
ATTGTTGAAAGGGAAACGATTGAAGTCAGACGTGCGTGATGCGGTTCAGCACTG
GTTCGCCAGTGTGTATTCCGTATCTTTGCAGGCCAACATCGGTTTCGATAGTCGGA
TAAAGATTAGTTGAATGTGGCACCTACGGGTGTGTTATAGCTTCTAATTGAATACG
ATTGTTGAGACCGAGGAACGCAGCGCGCCGCAAGGCAAAGGTTCCGACCTTTTC
GCGCTTAGGATGTTGGTGAAATGGCTTTAAACGAC

35. ***Cyberlindnera saturnus*** **SWU-NGP 1-5-2-1**

>SWU-NGP 1-5-2-1 (572 bp)

ACCAACAGGGATTGCCTCAGTAACGGCGAGTGAAGCGGCAAAAGCTCAAATTTG
AAATCTGGTACCTTTGGTGCCCGAGTTGTAATTTGAAGATAGTTTTCTGGTGCTGG
CCCTTGTCTATGTTCCTTGGAACAGGACGTCACAGAGGGTGAGAATCCCGTCTGG
CGGGGTGTCCAGTGCTTTGTAGATTTCTATCGACGAGTCGAGTTGTTTGGGAATGC
AGCTCTAAGTGGGTGGTAAATTCCATCTAAAGCTAAATATTGGCGAGAGACCGAT
AGCGAACAAGTACAGTGATGGAAAGATGAAAAGAACTTTGAAAAGAGAGTGAA
AAAGTACGTGAAATTGTTGAAAGGGAAGGGTATTAGATCAGACTTGGTGTTTTGT
GATTATCTTCCCTTCTTGGGTTGTGCACTCGCATTTCACTGGGCCAGCATCGGTTCG
GGTGGTAAGATAATGACATTGGAACGTGGCACTACCTTCGGGTGGTGTGTTATAG
CCCTTGTTGATGTTGCCTACCTGGACCGAGGACTGCGGCTTTTGCCTAGGATGCTG
GCGTAATGATCTAACACCGC

36. ***Papiliotrema flavescens*** **SWU-NGTP 2-5**

>SWU-NGTP 2-5 (592 bp)

CTAACAAGGATTCCCCTAGTAACGGCGAGTGAACCGGGAAGAGCTCAAATTTGA
AATCTGGCGTGCTCAGTGCGTCCGAGTTGTAATCTATAGAGTCGTTTTCCGTGCCG
GACTGTGTCCAAGTCCCTTGGAACAGGGTATCAAAGAGGGTGATAATCCCGTACT
TGACACAATGACCGGTGCTCTGTGATACGTCTTCTACGAGTCGAGTTGTTTGGGA

ATGCAGCTCAAAATGGGTGGTGAGTTCCATCTAAAGCTAAATATTGGCGAGAGA
CCGATAGCGAACAAGTACCGTGAGGGAAAGATGAAAAGCACTTTGGAAAGAGA
GTTAAACAGTACGTGAAATTGTTAAAAGGGAAACGATTGAAGTCAGTCGTGACT
GAGAGGCTCAGCCGGTTCTGCCGGTGTATTCCCCTCAGTCGGGTCAACATCAGTTT
TGTTCGGTGGATAAGGGCAGTTGGAAGGTGGCACCCTCGGGTGTGTTATAGCCAG
CTGTCGCATACATCGGATGAGACTGAGGAATGCAGCTCGCCTTTATGGCCGGGGT
TCGCCCACGTTCGAGCTTAGGATGTTGACATAATGGCTTTAAACG

37. ***Cyberlindnera saturnus*** **SWU-NGTP 4-14-2**
>SWU-NGTP 4-14-2 (470 bp)
AGTAACGGCGAGTGAAGCGGCAAAAGCTCAAATTTGAAATCTGGTACCTTTGGT
GCCCGAGTTGTAATTTGAAGATAGTTTTCTGGTGCTGGCCCTTGTCTATGTTCCTTG
GAACAGGACGTCACAGAGGGTGAGAATCCCGTCTGGCGGGGTGTCCAGTGCTTT
GTAGATTTCTATCGACGAGTCGAGTTGTTTGGGAATGCAGCTCTAAGTGGGTGGT
AAATTCCATCTAAAGCTAAATATTGGCGAGAGACCGATAGCGAACAAGTACAGT
GATGGAAAGATGAAAAGAACTTTGAAAAGAGAGTGAAAAAGTACGTGAAATTG
TTGAAAGGGAAGGGTATTAGATCAGACTTGGTGTTTTGTGATTATCTTCCCTTCTT
GGGTTGTGCACTCGCATTTCACTGGGCCAGCATCGGTTCGGGTGGTAAGATAATG
ACATTGGAACGTGGCACTACCTTCGGGTGGTG

38. ***Saitozyma podzolica*** **SWU-NGP 5-3-2**
>SWU-NGP 5-3-2 (588 bp)
CAAGGATTCCCTTAGTAGCGGCGAGCGAAGCGGGAAGAGCTCAAATTTGAAAGC
TGGCGTCCTACGGGCGTCCGCATTGTAATCTATAGAGGCGTTTTCTGTGCTGGACC
GTGTCCAAGTCCCTTGGAACAGGGTATCAAAGAGGGTGACAATCCCGTACTTGAC
ACGACAACCAGTGCTCTGTGATACGTCTTCTACGAGTCGAGTTGTTTGGGAATGC
AGCTCAAAATGGGTGGTGAATTCCATCTAAAGCTAAATATTGGCGAGAGACCGA
TAGCGAACAAGTACCGTGAGGGAAAGATGAAAAGCACTTTGGAAAGAGAGTTA
AACAGTATGTGAAATTGTTGAAAGGGAAACGATGGAGGTCAGTCGTGTCTGTGG
GATTCAGCCGTCTCTGGCGGTGTACTTCCCACAGACGGGTCAACATCAGTTTTGG
CTGGCGGATAAAGGCAGGAGGAAGGTGGCACCCCCGGGTGTGTTATAGCCTCTT
GTTGCATACGCTGGCTGAGACTGAGGAATGCAGCTCGCCTTTATGGCCGGGGTTC
GCCCACGTTCGAGCTTAGGATGTTGACATAATGGCCTTCAACG

39. ***Papiliotrema flavescens*** **SWU-NAP 3-2**

>SWU-NAP 3-2 (585 bp)

ATTCCCCTAGTAACGGCGAGTGAACCGGGAAGAGCTCAAATTTGAAATCTGGCG
TGCTCAGTGCGTCCGAGTTGTAATCTATAGAGTCGTTTTCCGTGCCGGACTGTGTC
CAAGTCCCTTGGAACAGGGTATCAAAGAGGGTGATAATCCCGTACTTGACACAA
TGACCGGTGCTCTGTGATACGTCTTCTACGAGTCGAGTTGTTTGGGAATGCAGCTC
AAAATGGGTGGTGAGTTCCATCTAAAGCTAAATATTGGCGAGAGACCGATAGCG
AACAAGTACCGTGAGGGAAAGATGAAAAGCACTTTGGAAAGAGAGTTAAACAG
TACGTGAAATTGTTAAAAGGGAAACGATTGAAGTCAGTCGTGACTGAGAGGCTC
AGCCGGTTCTGCCGGTGTATTCCCCTCAGTCGGGTCAACATCAGTTTTGTTCGGTG
GATAAGGGCAGTTGGAAGGTGGCACCCTCGGGTGTGTTATAGCCAGCTGTCGCAT
ACATCGGATGAGACTGAGGAATGCAGCTCGCCTTTATGGCCGGGGTTCGCCCACG
TTCGAGCTTAGGATGTTGACATAATGGCTTTAAACGAC

40. ***Lipomyces mesembrius*** **SWU-NAP 8-4**

>SWU-NAP 8-4 (571 bp)

AAACCAACAGGGATTGCCTTAGTAACTGCGAGTGAAGCGGCAAGAGCTCAAATT
TGAAATCTGGCACCTTCGGTGTCCGAGTTGTAATTTGTAGAAGCATCTTTTGGATT
GGTCCTTGCATATGTTCCTTGGAACAGGACATCATAGAGGGTGAGAATCCCGTTC
ATGGTGGGGAATCCAATTCTTTGTAAAGTGCTTTCGAAGAGTCGAGTTGTTTGGGA
ATGCAGCTCTAAGTGGGTGGTAAATTCCATCTAAAGCTAAATATTGGCGAGAGAC
CGATAGCGAACAAGTACAGTGATGGAAAGATGAAAAGAACTCCGAAAGGAGAG
TGAAAAAGGACGTGAAATTGTTGAAAGGGAAGTGCTTGAGATCAGTCTTAAGGT
TTAGTGATCAGTCTTCCTTTTGGTTGGTGCACTCACTATTTCTTAGGCCAACATCAG
TTTTGGCGGCAGGATAATGACATTGGAATGTGGCTCTTTCGGGAGTGTTATAGCCT
TTGTTGATACTGCCTGCTTAGACTGAGGACTGCGCTTTTGCTAGGATGTTGGCATA
ATGATCTTAAGCAGCCCGT

41. ***Saitozyma podzolica*** **SWU-NAP 5-4-2**

>SWU-NAP 5-4-2 (590 bp)

CAAGGATTCCCTTAGTAGCGGCGAGCGAAGCGGGAAGAGCTCAAATTTGAAAGC
TGGCGTCCTACGGGCGTCCGCATTGTAATCTATAGAGGCGTTTTCTGTGCTGGACC
GTGTCCAAGTCCCTTGGAACAGGGTATCAAAGAGGGTGACAATCCCGTACTTGAC
ACGACAACCAGTGCTCTGTGATACGTCTTCTACGAGTCGAGTTGTTTGGGAATGC

AGCTCAAAATGGGTGGTGAATTCCATCTAAAGCTAAATATTGGCGAGAGACCGA
TAGCGAACAAGTACCGTGAGGGAAAGATGAAAAGCACTTTGGAAAGAGAGTTA
AACAGTATGTGAAATTGTTGAAAGGGAAACGATGGAGGTCAGTCGTGTCTGTGG
GATTCAGCCGTCTCTGGCGGTGTACTTCCCACAGACGGGTCAACATCAGTTTTGG
CTGGCGGATAAAGGCAGGAGGAAGGTGGCACCCCCGGGTGTGTTATAGCCTCTT
GTTGCATACGCTGGCTGAGACTGAGGAATGCAGCTCGCCTTTATGGCCGGGGTTC
GCCCACGTTCGAGCTTAGGATGTTGACATAATGGCCTTCAACGAC

42. ***Papiliotrema flavescens*** **SWU-NATP 4-15**
> SWU-NATP 4-15 (576 bp)
CAAGGATTCCCCTAGTAACGGCGAGTGAACCGGGAAGAGCTCAAATTTGAAATC
TGGCGTGCTCAGTGCGTCCGAGTTGTAATCTATAGAGTCGTTTTCCGTGCCGGACT
GTGTCCAAGTCCCTTGGAACAGGGTATCAAAGAGGGTGATAATCCCGTACTTGAC
ACAATGACCGGTGCTCTGTGATACGTCTTCTACGAGTCGAGTTGTTTGGGAATGC
AGCTCAAAATGGGTGGTGAGTTCCATCTAAAGCTAAATATTGGCGAGAGACCGA
TAGCGAACAAGTACCGTGAGGGAAAGATGAAAAGCACTTTGGAAAGAGAGTTA
AACAGTACGTGAAATTGTTAAAAGGGAAACGATTGAAGTCAGTCGTGACTGAGA
GGCTCAGCCGGTTCTGCCGGTGTATTCCCCTCAGTCGGGTCAACATCAGTTTTGTT
CGGTGGATAAGGGCAGTTGGAAGGTGGCACCCTCGGGTGTGTTATAGCCAGCTGT
CGCATACATCGGATGAGACTGAGGAATGCAGCTCGCCTTTATGGCCGGGGTTCGC
CCACGTTCGAGCTTAGGATGTTGACATAA

43. ***Lipomyces tetrasporus*** **SWU-NAP 4-2-1**
>SWU-NAP 4-2-1 (562 bp)
ACCAACAGGGATTGCCTTAGTAACTGCGAGTGAAGCGGCAAGAGCTCAAATTTG
AAATCTGGCACCTTCGGTGTCCGAGTTGTAATTTGTAGAAGCATCTTTTGGATTGG
TCCTTGCATATGTTCCTTGGAACAGGACATCATAGAGGGTGAGAATCCCGTTCAT
GGTGGGGAATCCAATTCTTTGTAAAGTGCTTTCGAAGAGTCGAGTTGTTTGGGAAT
GCAGCTCTAAGTGGGTGGTAAATTCCATCTAAAGCTAAATATTGGCGAGAGACCG
ATAGCGAACAAGTACAGTGATGGAAAGATGAAAAGAACTCCGAAAGGAGAGTG
AAAAAGGACGTGAAATTGTTGAAAGGGAAGTGCTTGAGATCAGTCTTAAGGTTT
AGTGATCAGTCTTCCTTTTGGTTGGTGCACTCACTATTTCTTAGGCCAACATCAGTT
TTCGCGGCAGGATAATGACATTGGAATGTGGCTCTTTCGGGAGTGTTATAGCCTTT

GTTGATACTGCCTGCTTAGACTGAGGACCGCGCTTTTGCTAGGATGTTGGCATAAT
GATCTTAAGC

44. ***Lipomyces tetrasporus*** **SWU-NAP 5-3-1-1**
>SWU-NAP 5-3-1-1 (538 bp)
TGCGAGTGAAGCGGCAAGAGCTCAAATTTGAAATCTGGCACCTTCGGTGTCCGAG
TTGTAATTTGTAGAAGCATCTTTTGGATTGGTCCTTGCATATGTTCCTTGGAACAG
GACATCATAGAGGGTGAGAATCCCGTTCATGGTGGGGAATCCAATTCTTTGTAAA
GTGCTTTCGAAGAGTCGAGTTGTTTGGGAATGCAGCTCTAAGTGGGTGGTAAATT
CCATCTAAAGCTAAATATTGGCGAGAGACCGATAGCGAACAAGTACAGTGATGG
AAAGATGAAAAGAACTCCGAAAGGAGAGTGAAAAAGGACGTGAAATTGTTGAA
AGGGAAGTGCTTGAGATCAGTCTTAAGGTTTAGTGATCAGTCTTCCTTTTGGTTGG
TGCACTCACTATTTCTTAGGCCAACATCAGTTTTCGCGGCAGGATAATGACATTGG
AATGTGGCTCTTTCGGGAGTGTTATAGCCTTTGTTGATACTGCCTGCTTAGACTGA
GGACCGCGCTTTTGCTAGGATGTTGGCATAATGATCTTAAGC

45. ***Lipomyces mesembrius*** **SWU-NAP 4-13**
>SWU-NAP 4-13 (567 bp)
AAACCAACAGGGATTGCCTTAGTAACTGCGAGTGAAGCGGCAAGAGCTCAAATT
TGAAATCTGGCACCTTCGGTGTCCGAGTTGTAATTTGTAGAAGCATCTTTTGGATT
GGTCCTTGCATATGTTCCTTGGAACAGGACATCATAGAGGGTGAGAATCCCGTTC
ATGGTGGGGAATCCAATTCTTTGTAAAGTGCTTTCGAAGAGTCGAGTTGTTTGGGA
ATGCAGCTCTAAGTGGGTGGTAAATTCCATCTAAAGCTAAATATTGGCGAGAGAC
CGATAGCGAACAAGTACAGTGATGGAAAGATGAAAAGAACTCCGAAAGGAGAG
TGAAAAAGGACGTGAAATTGTTGAAAGGGAAGTGCTTGAGATCAGTCTTAAGGT
TTAGTGATCAGTCTTCCTTTTGGTTGGTGCACTCACTATTTCTTAGGCCAACATCAG
TTTTGGCGGCAGGATAATGACATTGGAATGTGGCTCTTTCGGGAGTGTTATAGCCT
TTGTTGATACTGCCTGCTTAGACTGAGGACTGCGCTTTTGCTAGGATGTTGGCATA
ATGATCTTAAGCAGC

46. ***Lipomyces mesembrius*** **SWU-NATP 1-4**
>SWU-NATP 1-4 (567 bp)
AAACCAACAGGGATTGCCTTAGTAACTGCGAGTGAAGCGGCAAGAGCTCAAATT
TGAAATCTGGCACCTTCGGTGTCCGAGTTGTAATTTGTAGAAGCATCTTTTGGATT

GGTCCTTGCATATGTTCCTTGGAACAGGACATCATAGAGGGTGAGAATCCCGTTC
ATGGTGGGGAATCCAATTCTTTGTAAAGTGCTTTCGAAGAGTCGAGTTGTTTGGGA
ATGCAGCTCTAAGTGGGTGGTAAATTCCATCTAAAGCTAAATATTGGCGAGAGAC
CGATAGCGAACAAGTACAGTGATGGAAAGATGAAAAGAACTCCGAAAGGAGAG
TGAAAAAGGACGTGAAATTGTTGAAAGGGAAGTGCTTGAGATCAGTCTTAAGGT
TTAGTGATCAGTCTTCCTTTTGGTTGGTGCACTCACTATTTCTTAGGCCAACATCAG
TTTTGGCGGCAGGATAATGACATTGGAATGTGGCTCTTTCGGGAGTGTTATAGCCT
TTGTTGATACTGCCTGCTTAGACTGAGGACTGCGCTTTTGCTAGGATGTTGGCATA
ATGATCTTAAGCAGC

47. ***Lipomyces mesembrius*** **SWU-NATP 4-11**
>SWU-NATP 4-11 (567 bp)
AAACCAACAGGGATTGCCTTAGTAACTGCGAGTGAAGCGGCAAGAGCTCAAATT
TGAAATCTGGCACCTTCGGTGTCCGAGTTGTAATTTGTAGAAGCATCTTTTGGATT
GGTCCTTGCATATGTTCCTTGGAACAGGACATCATAGAGGGTGAGAATCCCGTTC
ATGGTGGGGAATCCAATTCTTTGTAAAGTGCTTTCGAAGAGTCGAGTTGTTTGGGA
ATGCAGCTCTAAGTGGGTGGTAAATTCCATCTAAAGCTAAATATTGGCGAGAGAC
CGATAGCGAACAAGTACAGTGATGGAAAGATGAAAAGAACTCCGAAAGGAGAG
TGAAAAAGGACGTGAAATTGTTGAAAGGGAAGTGCTTGAGATCAGTCTTAAGGT
TTAGTGATCAGTCTTCCTTTTGGTTGGTGCACTCACTATTTCTTAGGCCAACATCAG
TTTTGGCGGCAGGATAATGACATTGGAATGTGGCTCTTTCGGGAGTGTTATAACCT
TTGTTGATACTGCCTGCTTATACTGACGACTGCGCTTTTGCTAGGATGTTGGCATA
ATGATCTTAAGCAGC

48. ***Hannaella*** **sp. SWU-YGP 11-1**
>SWU-YGP 11-1 (597 bp)
AAACTAACAAGGATTCCCCTAGTAGCGGCGAGCGAACCGGGAAGAGCTCAAATT
TAAAATCTGGCGTCTTTCAGGCGTCCGAGTTGTAATCTACAGAAGTGTTTTCCGTG
CCGGACCGTGTCCAAGTCCCTTGGAATAGGGTATCAAAGAGGGTGATAATCCCGT
ACTTGACACGACAACCGGTGCTCTGTGATACATTTTCTACGAGTCGAGTTGTTTGG
GAATGCAGCTCAAAATGGGTGGTAAATTCCATCTAAAGCTAAATATAGGCGAGA
GACCGATAGCGAACAAGTACCGTGAGGGAAAGATGAAAAGCACTTTGGAAAGA
GAGTTAAACAGTACGTGAAATTGTTAAAAGGGAAACGATTGAAGTCAGTCATGT
CTATTGGTTTCAGCTAGCTCTGCTAGTGTATTACCTTTAGACGGGTCAACATCAGT

TTTAGACGGTGGAAAAAGGCATGAGGAAAGTAGCAACTCCGGTTGTGTTATAGC
CTCGTGTTGCATACACTGTCCGAGACTGAGGAATGCAGCTCGCCTTTATGGCCGG
GGTTCGCCCACGTTCGAGCTTAGGATGTTGACATAATGGCTTTAAACGAC

49. ***Lipomyces mesembrius*** **SWU-NATP 4-8**
>SWU-NATP 4-8 (567 bp)
AAACCAACAGGGATTGCCTTAGTAACTGCGAGTGAAGCGGCAAGAGCTCAAATT
TGAAATCTGGCACCTTCGGTGTCCGAGTTGTAATTTGTAGAAGCATCTTTTGGATT
GGTCCTTGCATATGTTCCTTGGAACAGGACATCATAGAGGGTGAGAATCCCGTTC
ATGGTGGGGAATCCAATTCTTTGTAAAGTGCTTTCGAAGAGTCGAGTTGTTTGGGA
ATGCAGCTCTAAGTGGGTGGTAAATTCCATCTAAAGCTAAATATTGGCGAGAGAC
CGATAGCGAACAAGTACAGTGATGGAAAGATGAAAAGAACTCCGAAAGGAGAG
TGAAAAAGGACGTGAAATTGTTGAAAGGGAAGTGCTTGAGATCAGTCTTAAGGT
TTAGTGATCAGTCTTCCTTTTGGTTGGTGCACTCACTATTTCTTAGGCCAACATCAG
TTTTGGCGGCAGGATAATGGCATTGGAATGTGGCTCTTTCGGGAGTGTTATAGCCT
TTGTTGATACTGCCTGCTTAGACTGAGGACTGCGCTTTTGCTAGGATGTTGGCATA
ATGATCTTAAGCAGC

50. ***Lipomyces tetrasporus*** **SWU-NAP 5-3-1-2**
>SWU-NAP 5-3-1-2 (567 bp)
AAACCAACAGGGATTGCCTTAGTAACTGCGAGTGAAGCGGCAAGAGCTCAAATT
TGAAATCTGGCACCTTCGGTGTCCGAGTTGTAATTTGTAGAAGCATCTTTTGGATT
GGTCCTTGCATATGTTCCTTGGAACAGGACATCATAGAGGGTGAGAATCCCGTTC
ATGGTGGGGAATCCAATTCTTTGTAAAGTGCTTTCGAAGAGTCGAGTTGTTTGGGA
ATGCAGCTCTAAGTGGGTGGTAAATTCCATCTAAAGCTAAATATTGGCGAGAGAC
CGATAGCGAACAAGTACAGTGATGGAAAGATGAAAAGAACTCCGAAAGGAGAG
TGAAAAAGGACGTGAAATTGTTGAAAGGGAAGTGCTTGAGATCAGTCTTAAGGT
TTAGTGATCAGTCTTCCTTTTGGTTGGTGCACTCACTATTTCTTAGGCCAACATCAG
TTTTCGCGGCAGGATAATGACATTGGAATGTGGCTCTTTCGGGAGTGTTATAGCCT
TTGTTGATACTGCCTGCTTAGACTGAGGACCGCGCTTTTGCTAGGATGTTGGCATA
ATGATCTTAAGCAGC

51. ***Papiliotrema flavescens*** **SWU-YGPui 12-1**

>SWU-YGPui 12-1 (572 bp)

GGCGAGTGAACCGGGAAGAGCTCAAATTTGAAATCTGGCGTGCTCAGTGCGTCC
GAGTTGTAATCTATAGAGTCGTTTTCCGTGCCGGACTGTGTCCAAGTCCCTTGGAA
CAGGGTATCAAAGAGGGTGATAATCCCGTACTTGACACAATGACCGGTGCTCTGT
GATACGTCTTCTACGAGTCGAGTTGTTTGGGAATGCAGCTCAAAATGGGTGGTGA
GTTCCATCTAAAGCTAAATATTGGCGAGAGACCGATAGCGAACAAGTACCGTGA
GGGAAAGATGAAAAGCACTTTGGAAAGAGAGTTAAACAGTACGTGAAATTGTTA
AAAGGGAAACGATTGAAGTCAGTCGTGACTGAGAGGCTCAGCCGGTTCTGCCGG
TGTATTCCCCTCAGTCGGGTCAACATCAGTTTTGTTCGGTGGATAAGGGCAGTTGG
AAGGTGGCACCCTCGGGTGTGTTATAGCCAGCTGTCGCATACATCGGATGAGACT
GAGGAATGCAGCTCGCCTTTATGGCCGGGGTTCGCCCACGTTCGAGCTTAGGATG
TTGACATAATGGCTTTAAACGACC

52. ***Lipomyces tetrasporu*** **SWU-NATP 2-12**

>SWU-NATP 2-12 (557 bp)

CAGGGATTGCCTTAGTAACTGCGAGTGAAGCGGCAAGAGCTCAAATTTGAAATCT
GGCACCTTCGGTGTCCGAGTTGTAATTTGTAGAAGCATCTTTTGGATTGGTCCTTG
CATATGTTCCTTGGAACAGGACATCATAGAGGGTGAGAATCCCGTTCATGGTGGG
GAATCCAATTCTTTGTAAAGTGCTTTCGAAGAGTCGAGTTGTTTGGGAATGCAGCT
CTAAGTGGGTGGTAAATTCCATCTAAAGCTAAATATTGGCGAGAGACCGATAGC
GAACAAGTACAGTGATGGAAAGATGAAAAGAACTCCGAAAGGAGAGTGAAAAA
GGACGTGAAATTGTTGAAAGGGAAGTGCTTGAGATCAGTCTTAAGGTTTAGTGAT
CAGTCTTCCTTTTGGTTGGTGCACTCACTATTTCTTAGGCCAACATCAGTTTTCGCG
GCAGGATAATGACATTGGAATGTGGCTCTTTCGGGAGTGTTATAGCCTTTGTTGAT
ACTGCCTGCTTAGACTGAGGACCGCGCTTTTGCTAGGATGTTGGCATAATGATCTT
AAGC

53. ***Lipomyces starkeyi*** **SWU-NAP 2-2**

>SWU-NAP 2-2 (563 bp)

ACCAACAGGGATTGCCTTAGTAACTGCGAGTGAAGCGGCAAGAGCTCAAATTTG
AAATCTGGCACCTTCGGTGTCCGAGTTGTAATTTGTAGAAGCATCTTTTGGATTGG
TCCTTGCATATGTTCCTTGGAACAGGACATCATAGAGGGTGAGAATCCCGTTCAT
GGTGGGGAATCCAATTCTTTGTAAAGTGCTTTCGAAGAGTCGAGTTGTTTGGGAAT

GCAGCTCTAAGTGGGTGGTAAATTCCATCTAAAGCTAAATATTGGCGAGAGACCG
ATAGCGAACAAGTACAGTGATGGAAAGATGAAAAGAACTCCGAAAGGAGAGTG
AAAAAGGACGTGAAATTGTTGAAAGGGAAGTGCTTGAGATCAGTCTTAAGGTTT
AGTGATCAGTCTTCCTTTTGGTTGGTGCACTCACTATTTCTTAGGCCAACATCAGTT
TTGGCGGCAGGATAATGACATTGGAATGTGGCTCTTTCGGGAGTGTTATAGCCTTT
GTTGATACTGCCTGCTTAGACTGAGGACTGCGCTTTTGCTAGGATGTTGGCATAAT
GATCTTAAGCA

54. ***Papiliotrema flavescens*** **SWU-NAP 1-4**
>SWU-NAP 1-4 (594 bp)
AACAAGGATTCCCCTAGTAACGGCGAGTGAACCGGGAAGAGCTCAAATTTGAAA
TCTGGCGTGCTCAGTGCGTCCGAGTTGTAATCTATAGAGTCGTTTTCCGTGCCGGA
CTGTGTCCAAGTCCCTTGGAACAGGGTATCAAAGAGGGTGATAATCCCGTACTTG
ACACAATGACCGGTGCTCTGTGATACGTCTTCTACGAGTCGAGTTGTTTGGGAAT
GCAGCTCAAAATGGGTGGTGAGTTCCATCTAAAGCTAAATATTGGCGAGAGACC
GATAGCGAACAAGTACCGTGAGGGAAAGATGAAAAGCACTTTGGAAAGAGAGT
TAAACAGTACGTGAAATTGTTAAAAGGGAAACGATTGAAGTCAGTCGTGACTGA
GAGGCTCAGCCGGTTCTGCCGGTGTATTCCCCTCAGTCGGGTCAACATCAGTTTTG
TTCGGTGGATAAGGGCAGTTGGAAGGTGGCACCCTCGGGTGTGTTATAGCCAGCT
GTCGCATACATCGGATGAGACTGAGGAATGCAGCTCGCCTTTATGGCCGGGGTTC
GCCCACGTTCGAGCTTAGGATGTTGACATAATGGCTTTAAACGACCC

55. ***Lipomyces tetrasporus*** **SWU-NGP 6-5**
>SWU-NGP 6-5 (565 bp)
AAACCAACAGGGATTGCCTTAGTAACTGCGAGTGAAGCGGCAAGAGCTCAAATT
TGAAATCTGGCACCTTCGGTGTCCGAGTTGTAATTTGTAGAAGCATCTTTTGGATT
GGTCCTTGCATATGTTCCTTGGAACAGGACATCATAGAGGGTGAGAATCCCGTTC
ATGGTGGGGAATCCAATTCTTTGTAAAGTGCTTTCGAAGAGTCGAGTTGTTTGGGA
ATGCAGCTCTAAGTGGGTGGTAAATTCCATCTAAAGCTAAATATTGGCGAGAGAC
CGATAGCGAACAAGTACAGTGATGGAAAGATGAAAAGAACTCCGAAAGGAGAG
TGAAAAAGGACGTGAAATTGTTGAAAGGGAAGTGCTTGAGATCAGTCTTAAGGT
TTAGTGATCAGTCTTCCTTTTGGTTGGTGCACTCACTATTTCTTAGGCCAACATCAG
TTTTCGCGGCAGGATAATGACATTGGAATGTGGCTCTTTCGGGAGTGTTATAGCCT

TTGTTGATACTGCCTGCTTAGACTGAGGACCGCGCTTTTGCTAGGATGTTGGCATA
ATGATCTTAAGCA

56. ***Lipomyces tetrasporus*** **SWU-NAP 2-3**

>SWU-NAP 2-3 (548 bp)
AAACCAACAGGGATTGCCTTAGTAACTGCGAGTGAAGCGGCAAGAGCTCAAATT
TGAAATCTGGCACCTTCGGTGTCCGAGTTGTAATTTGTAGAAGCATCTTTTGGATT
GGTCCTTGCATATGTTCCTTGGAACAGGACATCATAGAGGGTGAGAATCCCGTTC
ATGGTGGGGAATCCAATTCTTTGTAAAGTGCTTTCGAAGAGTCGAGTTGTTTGGGA
ATGCAGCTCTAAGTGGGTGGTAAATTCCATCTAAAGCTAAATATTGGCGAGAGAC
CGATAGCGAACAAGTACAGTGATGGAAAGATGAAAAGAACTCCGAAAGGAGAG
TGAAAAAGGACGTGAAATTGTTGAAAGGGAAGTGCTTGAGATCAGTCTTAAGGT
TTAGTGATCAGTCTTCCTTTTGGTTGGTGCACTCACTATTTCTTAGGCCAACATCAG
TTTTCGCGGCAGGATAATGACATTGGAATGTGGCTCTTTCGGGAGTGTTATAGCCT
TTGTTGATACTGCCTGCTTAGACTGAGGACCGCGCTTTTGCTAGGATGTTGG

57. ***Lipomyces tetrasporus*** **SWU-NAP 5-5**

>SWU-NAP 5-5 (564 bp)
CCAACAGGGATTGCCTTAGTAACTGCGAGTGAAGCGGCAAGAGCTCAAATTTGA
AATCTGGCACCTTCGGTGTCCGAGTTGTAATTTGTAGAAGCATCTTTTGGATTGGT
CCTTGCATATGTTCCTTGGAACAGGACATCATAGAGGGTGAGAATCCCGTTCATG
GTGGGGAATCCAATTCTTTGTAAAGTGCTTTCGAAGAGTCGAGTTGTTTGGGAATG
CAGCTCTAAGTGGGTGGTAAATTCCATCTAAAGCTAAATATTGGCGAGAGACCGA
TAGCGAACAAGTACAGTGATGGAAAGATGAAAAGAACTCCGAAAGGAGAGTGA
AAAAGGACGTGAAATTGTTGAAAGGGAAGTGCTTGAGATCAGTCTTAAGGTTTA
GTGATCAGTCTTCCTTTTGGTTGGTGCACTCACTATTTCTTAGGCCAACATCAGTTT
TCGCGGCAGGATAATGACATTGGAATGTGGCTCTTTCGGGAGTGTTATAGCCTTTG
TTGATACTGCCTGCTTAGACTGAGGACCGCGCTTTTGCTAGGATGTTGGCATAATG
ATCTTAAGCAGC

58. ***Lipomyces tetrasporus*** **SWU-NAP 5-6**

>SWU-NAP 5-6 (562 bp)
AACAGGGATTGCCTTAGTAACTGCGAGTGAAGCGGCAAGAGCTCAAATTTGAAA
TCTGGCACCTTCGGTGTCCGAGTTGTAATTTGTAGAAGCATCTTTTGGATTGGTCCT

TGCATATGTTCCTTGGAACAGGACATCATAGAGGGTGAGAATCCCGTTCATGGTG
GGGAATCCAATTCTTTGTAAAGTGCTTTCGAAGAGTCGAGTTGTTTGGGAATGCA
GCTCTAAGTGGGTGGTAAATTCCATCTAAAGCTAAATATTGGCGAGAGACCGATA
GCGAACAAGTACAGTGATGGAAAGATGAAAAGAACTCCGAAAGGAGAGTGAAA
AAGGACGTGAAATTGTTGAAAGGGAAGTGCTTGAGATCAGTCTTAAGGTTTAGTG
ATCAGTCTTCCTTTTGGTTGGTGCACTCACTATTTCTTAGGCCAACATCAGTTTTCG
CGGCAGGATAATGACATTGGAATGTGGCTCTTTCGGGAGTGTTATAGCCTTTGTTG
ATACTGCCTGCTTAGACTGAGGACCGCGCTTTTGCTAGGATGTTGGCATAATGATC
TTAAGCAGC

59. ***Lipomyces tetrasporus*** **SWU-NAP 5-2-2**

>SWU-NAP 5-2-2 (556 bp)
GATTGCCTTAGTAACTGCGAGTGAAGCGGCAAGAGCTCAAATTTGAAATCTGGCA
CCTTCGGTGTCCGAGTTGTAATTTGTAGAAGCATCTTTTGGATTGGTCCTTGCATAT
GTTCCTTGGAACAGGACATCATAGAGGGTGAGAATCCCGTTCATGGTGGGGAATC
CAATTCTTTGTAAAGTGCTTTCGAAGAGTCGAGTTGTTTGGGAATGCAGCTCTAAG
TGGGTGGTAAATTCCATCTAAAGCTAAATATTGGCGAGAGACCGATAGCGAACA
AGTACAGTGATGGAAAGATGAAAAGAACTCCGAAAGGAGAGTGAAAAAGGACG
TGAAATTGTTGAAAGGGAAGTGCTTGAGATCAGTCTTAAGGTTTAGTGATCAGTC
TTCCTTTTGGTTGGTGCACTCACTATTTCTTAGGCCAACATCAGTTTTCGCGGCAGG
ATAATGACATTGGAATGTGGCTCTTTCGGGAGTGTTATAGCCTTTGTTGATACTGC
CTGCTTAGACTGAGGACCGCGCTTTTGCTAGGATGTTGGCATAATGATCTTAAGC
AGC

60. ***Lipomyces starkeyi*** **SWU-NATP 4-2**

>SWU-NATP 4-2 (556 bp)
GATTGCCTTAGTAACTGCGAGTGAAGCGGCAAGAGCTCAAATTTGAAATCTGGCA
CCTTCGGTGTCCGAGTTGTAATTTGTAGAAGCATCTTTTGGATTGGTCCTTGCATAT
GTTCCTTGGAACAGGACATCATAGAGGGTGAGAATCCCGTTCATGGTGGGGAATC
CAATTCTTTGTAAAGTGCTTTCGAAGAGTCGAGTTGTTTGGGAATGCAGCTCTAAG
TGGGTGGTAAATTCCATCTAAAGCTAAATATTGGCGAGAGACCGATAGCGAACA
AGTACAGTGATGGAAAGATGAAAAGAACTCCGAAAGGAGAGTGAAAAAGGACG
TGAAATTGTTGAAAGGGAAGTGCTTGAGATCAGTCTTAAGGTTTAGTGATCAGTC
TTCCTTTTGGTTGGTGCACTCACTATTTCTTAGGCCAACATCAGTTTTGGCGGCAG

GATAATGACATTGGAATGTGGCTCTTTCGAGAGTGTTATAGCCTTTGTTGATACTG
CCTGCTTAGACTGAGGACTGCGCTTTTGCTAGGATGTTGGCATAATGATCTTAAGC
AGC

61. ***Lipomyces tetrasporus*** **SWU-NAP 5-2-1**

>SWU-NAP 5-2-1 (551 bp)
TTGCCTTAGTAACTGCGAGTGAAGCGGCAAGAGCTCAAATTTGAAATCTGGCACC
TTCGGTGTCCGAGTTGTAATTTGTAGAAGCATCTTTTGGATTGGTCCTTGCATATGT
TCCTTGGAACAGGACATCATAGAGGGTGAGAATCCCGTTCATGGTGGGGAATCC
AATTCTTTGTAAAGTGCTTTCGAAGAGTCGAGTTGTTTGGGAATGCAGCTCTAAGT
GGGTGGTAAATTCCATCTAAAGCTAAATATTGGCGAGAGACCGATAGCGAACAA
GTACAGTGATGGAAAGATGAAAAGAACTCCGAAAGGAGAGTGAAAAAGGACGT
GAAATTGTTGAAAGGGAAGTGCTTGAGATCAGTCTTAAGGTTTAGTGATCAGTCT
TCCTTTTGGTTGGTGCACTCACTATTTCTTAGGCCAACATCAGTTTTCGCGGCAGG
ATAATGACATTGGAATGTGGCTCTTTCGGGAGTGTTATAGCCTTTGTTGATACTGC
CTGCTTAGACTGAGGACCGCGCTTTTGCTAGGATGTTGGCATAATGATCTTAAGC

62. ***Lipomyces tetrasporus*** **SWU-NATP 9-2**

>SWU-NATP 9-2 (552 bp)
TTGCCTTAGTAACTGCGAGTGAAGCGGCAAGAGCTCAAATTTGAAATCTGGCACC
TTCGGTGTCCGAGTTGTAATTTGTAGAAGCATCTTTTGGATTGGTCCTTGCATATGT
TCCTTGGAACAGGACATCATAGAGGGTGAGAATCCCGTTCATGGTGGGGAATCC
AATTCTTTGTAAAGTGCTTTCGAAGAGTCGAGTTGTTTGGGAATGCAGCTCTAAGT
GGGTGGTAAATTCCATCTAAAGCTAAATATTGGCGAGAGACCGATAGCGAACAA
GTACAGTGATGGAAAGATGAAAAGAACTCCGAAAGGAGAGTGAAAAAGGACGT
GAAATTGTTGAAAGGGAAGTGCTTGAGATCAGTCTTAAGGTTTAGTGATCAGTCT
TCCTTTTGGTTGGTGCACTCACTATTTCTTAGGCCAACATCAGTTTTCGCGGCAGG
ATAATGACATTGGAATGTGGCTCTTTCGGGAGTGTTATAGCCTTTGTTGATACTGC
CTGCTTAGACTGAGGACCGCGCTTTTGCTAGGATGTTGGCATAATGATCTTAAGC
A

63. ***Lipomyces tetrasporus*** **SWU-NAP 5-3-3**

>SWU-NAP 5-3-3 (549 bp)

CCTTAGTAACTGCGAGTGAAGCGGCAAGAGCTCAAATTTGAAATCTGGCACCTTC
GGTGTCCGAGTTGTAATTTGTAGAAGCATCTTTTGGATTGGTCCTTGCATATGTTCC
TTGGAACAGGACATCATAGAGGGTGAGAATCCCGTTCATGGTGGGGAATCCAAT
TCTTTGTAAAGTGCTTTCGAAGAGTCGAGTTGTTTGGGAATGCAGCTCTAAGTGGG
TGGTAAATTCCATCTAAAGCTAAATATTGGCGAGAGACCGATAGCGAACAAGTA
CAGTGATGGAAAGATGAAAAGAACTCCGAAAGGAGAGTGAAAAAGGACGTGAA
ATTGTTGAAAGGGAAGTGCTTGAGATCAGTCTTAAGGTTTAGTGATCAGTCTTCCT
TTTGGTTGGTGCACTCACTATTTCTTAGGCCAACATCAGTTTTCGCGGCAGGATAA
TGACATTGGAATGTGGCTCTTTCGGGAGTGTTATAGCCTTTGTTGATACTGCCTGC
TTAGACTGAGGACCGCGCTTTTGCTAGGATGTTGGCATAATGATCTTAAGCA

64. ***Lipomyces tetrasporus*** **SWU-NAP 6-1**

>SWU-NAP 6-1 (545 bp)

GCCTTAGTAACTGCGAGTGAAGCGGCAAGAGCTCAAATTTGAAATCTGGCACCTT
CGGTGTCCGAGTTGTAATTTGTAGAAGCATCTTTTGGATTGGTCCTTGCATATGTTC
CTTGGAACAGGACATCATAGAGGGTGAGAATCCCGTTCATGGTGGGGAATCCAA
TTCTTTGTAAAGTGCTTTCGAAGAGTCGAGTTGTTTGGGAATGCAGCTCTAAGTGG
GTGGTAAATTCCATCTAAAGCTAAATATTGGCGAGAGACCGATAGCGAACAAGT
ACAGTGATGGAAAGATGAAAAGAACTCCGAAAGGAGAGTGAAAAAGGACGTGA
AATTGTTGAAAGGGAAGTGCTTGAGATCAGTCTTAAGGTTTAGTGATCAGTCTTCC
TTTTGGTTGGTGCACTCACTATTTCTTAGGCCAACATCAGTTTTCGCGGCAGGATA
ATGACATTGGAATGTGGCTCTTTCGGGAGTGTTATAGCCTTTGTTGATACTGCCTG
CTTAGACTGAGGACCGCGCTTTTGCTAGGATGTTGGCATAATGATCTT

65. ***Lipomyces tetrasporus*** **SWU-NAP 13-7**

>SWU-NAP 13-7 (536 bp)

TAGTAACTGCGAGTGAAGCGGCAAGAGCTCAAATTTGAAATCTGGCACCTTCGGT
GTCCGAGTTGTAATTTGTAGAAGCATCTTTTGGATTGGTCCTTGCATATGTTCCTTG
GAACAGGACATCATAGAGGGTGAGAATCCCGTTCATGGTGGGGAATCCAATTCTT
TGTAAAGTGCTTTCGAAGAGTCGAGTTGTTTGGGAATGCAGCTCTAAGTGGGTGG
TAAATTCCATCTAAAGCTAAATATTGGCGAGAGACCGATAGCGAACAAGTACAG
TGATGGAAAGATGAAAAGAACTCCGAAAGGAGAGTGAAAAAGGACGTGAAATT

GTTGAAAGGGAAGTGCTTGAGATCAGTCTTAAGGTTTAGTGATCAGTCTTCCTTTT
GGTTGGTGCACTCACTATTTCTTAGGCCAACATCAGTTTTCGCGGCAGGATAATGA
CATTGGAATGTGGCTCTTTCGGGAGTGTTATAGCCTTTGTTGATACTGCCTGCTTA
GACTGAGGACCGCGCTTTTGCTAGGATGTTGGCATAATG

66. ***Lipomyces starkeyi*** **SWU-NATP 1-1**

>SWU-NATP 1-1 (546 bp)
GATTGCCTTAGTAACTGCGAGTGAAGCGGCAAGAGCTCAAATTTGAAATCTGGCA
CCTTCGGTGTCCGAGTTGTAATTTGTAGAAGCATCTTTTGGATTGGTCCTTGCATAT
GTTCCTTGGAACAGGACATCATAGAGGGTGAGAATCCCGTTCATGGTGGGGAATC
CAATTCTTTGTAAAGTGCTTTCGAAGAGTCGAGTTGTTTGGGAATGCAGCTCTAAG
TGGGTGGTAAATTCCATCTAAAGCTAAATATTGGCGAGAGACCGATAGCGAACA
AGTACAGTGATGGAAAGATGAAAAGAACTCCGAAAGGAGAGTGAAAAAGGACG
TGAAATTGTTGAAAGGGAAGTGCTTGAGATCAGTCTTAAGGTTTAGTGATCAGTC
TTCCTTTTGGTTGGTGCACTCACTATTTCTTAGGCCAACATCAGTTTTGGCGGCAG
GATAATGACATTGGAATGTGGCTCTTTCGGGAGTGTTATAGCCTTTGTTGATACTG
CCTGCTTAGACTGAGGACTGCGCTTTTGCTAGGATGTTGGCATAATGAT

67. ***Lipomyces starkeyi*** **SWU-NATP 4-5**

>SWU-NATP 4-5 (544 bp)
TGCCTTAGTAACTGCGAGTGAAGCGGCAAGAGCTCAAATTTGAAATCTGGCACCT
TCGGTGTCCGAGTTGTAATTTGTAGAAGCATCTTTTGGATTGGTCCTTGCATATGTT
CCTTGGAACAGGACATCATAGAGGGTGAGAATCCCGTTCATGGTGGGGAATCCA
ATTCTTTGTAAAGTGCTTTCGAAGAGTCGAGTTGTTTGGGAATGCAGCTCTAAGTG
GGTGGTAAATTCCATCTAAAGCTAAATATTGGCGAGAGACCGATAGCGAACAAG
TACAGTGATGGAAAGATGAAAAGAACTCCGAAAGGAGAGTGAAAAAGGACGTG
AAATTGTTGAAAGGGAAGTGCTTGAGATCAGTCTTAAGGTTTAGTGATCAGTCTT
CCTTTTGGTTGGTGCACTCACTATTTCTTAGGCCAACATCAGTTTTGGCGGCAGGA
TAATGACATTGGAATGTGGCTCTTTCGGGAGTGTTATAGCCTTTGTTGATACTGCC
TGCTTAGACTGAGGACTGCGCTTTTGCTAGGATGTTGGCATAATGATC

68. ***Lipomyces starkeyi*** **SWU-NAP 10-1**

>SWU-NAP 10-1 (553 bp)

GGATTGCCTTAGTAACTGCGAGTGAAGCGGCAAGAGCTCAAATTTGAAATCTGGC
ACCTTCGGTGTCCGAGTTGTAATTTGTAGAAGCATCTTTTGGATTGGTCCTTGCAT
ATGTTCCTTGGAACAGGACATCATAGAGGGTGAGAATCCCGTTCATGGTGGGGA
ATCCAATTCTTTGTAAAGTGCTTTCGAAGAGTCGAGTTGTTTGGGAATGCAGCTCT
AAGTGGGTGGTAAATTCCATCTAAAGCTAAATATTGGCGAGAGACCGATAGCGA
ACAAGTACAGTGATGGAAAGATGAAAAGAACTCCGAAAGGAGAGTGAAAAAGG
ACGTGAAATTGTTGAAAGGGAAGTGCTTGAGATCAGTCTTAAGGTTTAGTGATCA
GTCTTCCTTTTGGTTGGTGCACTCACTATTTCTTAGGCCAACATCAGTTTTGGCGGC
AGGATAATGACATTGGAATGTGGCTCTTTCGGGAGTGTTATAGCCTTTGTTGATAC
TGCCTGCTTAGACTGAGGACTGCGCTTTTGCTAGGATGTTGGCATAATGATCTTAA
G

69. ***Lipomyces tetrasporus*** **SWU-NAP 5-1-2**

>SWU-NAP 5-1-2 (546 bp)

AGGGATTGCCTTAGTAACTGCGAGTGAAGCGGCAAGAGCTCAAATTTGAAATCT
GGCACCTTCGGTGTCCGAGTTGTAATTTGTAGAAGCATCTTTTGGATTGGTCCTTG
CATATGTTCCTTGGAACAGGACATCATAGAGGGTGAGAATCCCGTTCATGGTGGG
GAATCCAATTCTTTGTAAAGTGCTTTCGAAGAGTCGAGTTGTTTGGGAATGCAGCT
CTAAGTGGGTGGTAAATTCCATCTAAAGCTAAATATTGGCGAGAGACCGATAGC
GAACAAGTACAGTGATGGAAAGATGAAAAGAACTCCGAAAGGAGAGTGAAAAA
GGACGTGAAATTGTTGAAAGGGAAGTGCTTGAGATCAGTCTTAAGGTTTAGTGAT
CAGTCTTCCTTTTGGTTGGTGCACTCACTATTTCTTAGGCCAACATCAGTTTTCGCG
GCAGGATAATGACATTGGAATGTGGCTCTTTCGGGAGTGTTATAGCCTTTGTTGAT
ACTGCCTGCTTAGACTGAGGACCGCGCTTTTGCTAGGATGTTGGCATAAT

70. ***Lipomyces starkeyi*** **SWU-NAPui 2-1**

>SWU-NAPui 2-1 (553 bp)

TTGCCTTAGTAACTGCGAGTGAAGCGGCAAGAGCTCAAATTTGAAATCTGGCACC
TTCGGTGTCCGAGTTGTAATTTGTAGAAGCATCTTTTGGATTGGTCCTTGCATATGT
TCCTTGGAACAGGACATCATAGAGGGTGAGAATCCCGTTCATGGTGGGGAATCC
AATTCTCTGTAAAGTGCTTTCGAAGAGTCGAGTTGTTTGGGAATGCAGCTCTAAGT
GGGTGGTAAATTCCATCTAAAGCTAAATATTGGCGAGAGACCGATAGCGAACAA

GTACAGTGATGGAAAGATGAAAAGAACTCCGAAAGGAGAGTGAAAAAGGACGT
GAAATTGTTGAAAGGGAAGTGCTTGAGATCAGTCTTAAGGTTTAGTGATCAGTCT
TCCTTTTGGTTGGTGCACTCACTATTTCTTAGGCCAACATCAGTTTTGGCGGCAGG
ATAATGACATTGGAATGTGGCTCTTTCGGGAGTGTTATAGCCTTTGTTGATACTGC
CTGCTTAGACTGAGGACTGCGCTTTTGCTAGGATGTTGGCATAATGATCTTA
GATCT

71. ***Lipomyces starkeyi*** **SWU-NATP 5-10-2**

>SWU-NATP 5-10-2 (549 bp)
TGCCTTAGTAACTGCGAGTGAAGCGGCAAGAGCTCAAATTTGAAATCTGGCACCT
TCGGTGTCCGAGTTGTAATTTGTAGAAGCATCTTTTGGATTGGTCCTTGCATATGTT
CCTTGGAACAGGACATCATAGAGGGTGAGAATCCCGTTCATGGTGGGGAATCCA
ATTCTTTGTAAAGTGCTTTCGAAGAGTCGAGTTGTTTGGGAATGCAGCTCTAAGTG
GGTGGTAAATTCCATCTAAAGCTAAATATTGGCGAGAGACCGATAGCGAACAAG
TACAGTGATGGAAAGATGAAAAGAACTCCGAAAGGAGAGTGAAAAAGGACGTG
AAATTGTTGAAAGGGAAGTGCTTGAGATCAGTCTTAAGGTTTAGTGATCAGTCTT
CCTTTTGGTTGGTGCACTCACTATTTCTTAGGCCAACATCAGTTTTGGCGGCAGGA
TAATGACATTGGAATGTGGCTCTTTCGGGAGTGTTATAGCCTTTGTTGATACTGCT
TGCTTAGACTGAGGACTGCGCTTTTGCTAGGATGTTGGCATAATGATCTTAAG

72. ***Saitozyma podzolica*** **SWU-NGP 5-10-2**

>SWU-NGP 5-10-2 (574 bp)
CAAGGATTCCCTTAGTAGCGGCGAGCGAAGCGGGAAGAGCTCAAATTTGAAAGC
TGGCGTCCTACGGGCGTCCGCATTGTAATCTATAGAGGCGTTTTCTGTGCTGGACC
GTGTCCAAGTCCCTTGGAACAGGGTATCAAAGAGGGTGACAATCCCGTACTTGAC
ACGACAACCAGTGCTCTGTGATACGTCTTCTACGAGTCGAGTTGTTTGGGAATGC
AGCTCAAAATGGGTGGTGAATTCCATCTAAAGCTAAATATTGGCGAGAGACCGA
TAGCGAACAAGTACCGTGAGGGAAAGATGAAAAGCACTTTGGAAAGAGAGTTA
AACAGTATGTGAAATTGTTGAAAGGGAAACGATGGAGGTCAGTCGTGTCTGTGG
GATTCAGCCGTCTCTGGCGGTGTACTTCCCACAGACGGGTCAACATCAGTTTTGG
CTGGCGGATAAAGGCAGGAGGAAGGTGGCACCCCCGGGTGTGTTATAGCCTCTT
GTTGCATACGCTGGCTGAGACTGAGGAATGCAGCTCGCCTTTATGGCCGGGGTTC
GCCCACGTTCGAGCTTAGGATGTTGACAT

73. ***Papiliotrema terrestris*** **SWU-NAP 12-1-12**

>SWU-NAP 12-1-12 (562 bp)

CTAGTAACGGCGAGTGAACCGGGAAGAGCTCAAATTTGAAATCTGGCGTGCTCA
GTGCGTCCGAGTTGTAATCTATAGAGGCGTTTTCCGTGCCGGACTGTGTCTAAGTC
CCTTGGAACAGGGTATCAAAGAGGGTGATAATCCCGTACTTGACACAATGACCG
GTGCTCTGTGATACGTCTTCTACGAGTCGAGTTGTTTGGGAATGCAGCTCAAAATG
GGTGGTGAGTTCCATCTAAAGCTAAATATTGGCGAGAGACCGATAGCGAACAAG
TACCGTGAGGGAAAGATGAAAAGCACTTTGGAAAGAGAGTTAAACAGTACGTGA
AATTGTTGAAAGGGAAACGATTGAAGTCAGTCGTGACTGAGAGGCTCAGCCGGT
TCTGCCGGTGTATTCCCCTCAGTCGGGTCAACATCAGTTTTGTTCGGTGGATAAGG
GCAGCTGGAAGGTGGCACCTCCGGGTGTGTTATAGCCAGCTGTCGCATACATCGA
ATGAGACTGAGGAATGCAGCTCGCCTTTATGGCCGGGGTTCGCCCACGTCCGAGC
TTAGGATGTTGACA

74. ***Papiliotrema terrestris*** **SWU-NAP 12-1-1**

>SWU-NAP 12-1-1 (579 bp)

GGATTCCCCTAGTAACGGCGAGTGAACCGGGAAGAGCTCAAATTTGAAATCTGG
CGTGCTCAGTGCGTCCGAGTTGTAATCTATAGAGGCGTTTTCCGTGCCGGACTGTG
TCTAAGTCCCTTGGAACAGGGTATCAAAGAGGGTGATAATCCCGTACTTGACACA
ATGACCGGTGCTCTGTGATACGTCTTCTACGAGTCGAGTTGTTTGGGAATGCAGCT
CAAAATGGGTGGTGAGTTCCATCTAAAGCTAAATATTGGCGAGAGACCGATAGC
GAACAAGTACCGTGAGGGAAAGATGAAAAGCACTTTGGAAAGAGAGTTAAACA
GTACGTGAAATTGTTGAAAGGGAAACGATTGAAGTCAGTCGTGACTGAGAGGCT
CAGCCGGTTCTGCCGGTGTATTCCCCTCAGTCGGGTCAACATCAGTTTTGTTCGGT
GGATAAGGGCAGCTGGAAGGTGGCACCTCCGGGTGTGTTATAGCCAGCTGTCGC
ATACATCGAATGAGACTGAGGAATGCAGCTCGCCTTTATGGCCGGGGTTCGCCCA
CGTCCGAGCTTAGGATGTTGACATAATGGCTT

75. ***Lipomyces starkeyi*** **SWU-NGTP 4-4**

>SWU-NGTP 4-4 (548 bp)

TTGCCTTAGTAACTGCGAGTGAAGCGGCAAGAGCTCAAATTTGAAATCTGGCACC
TTCGGTGTCCGAGTTGTAATTTGTAGAAGCATCTTTTGGATTGGTCCTTGCATATGT
TCCTTGGAACAGGACATCATAGAGGGTGAGAATCCCGTTCATGGTGGGGAATCC
AATTCTTTGTAAAGTGCTTTCGAAGAGTCGAGTTGTTTGGGAATGCAGCTCTAAGT

GGGTGGTAAATTCCATCTAAAGCTAAATATTGGCGAGAGACCGATAGCGAACAA
GTACAGTGATGGAAAGATGAAAAGAACTCCGAAAGGAGAGTGAAAAAGGACGT
GAAATTGTTGAAAGGGAAGTGCTTGAGATCAGTCTTAAGGTTTAGTGATCAGTCT
TCCTTTTGGTTGGTGCACTCACTATTTCTTAGGCCAACATCAGTTTTGGCGGCAGG
ATAATGACATTGGAATGTGGCTCTTTCGGGAGTGTTATAGCCTTTGTTGATACTGC
CTGCTTAGACAGAGGACTGCGCTTTTGCTAGGATGTTGGCATAATGATCTTA

76. ***Lipomyces tetrasporus*** **SWU-NGP 3-6**

>SWU-NGP 3-6 (550 bp)
TTGCCTTAGTAACTGCGAGTGAAGCGGCAAGAGCTCAAATTTGAAATCTGGCACC
TTCGGTGTCCGAGTTGTAATTTGTAGAAGCATCTTTTGGATTGGTCCTTGCATATGT
TCCTTGGAACAGGACATCATAGAGGGTGAGAATCCCGTTCATGGTGGGGAATCC
AATTCTTTGTAAAGTGCTTTCGAAGAGTCGAGTTGTTTGGGAATGCAGCTCTAAGT
GGGTGGTAAATTCCATCTAAAGCTAAATATTGGCGAGAGACCGATAGCGAACAA
GTACAGTGATGGAAAGATGAAAAGAACTCCGAAAGGAGAGTGAAAAAGGACGT
GAAATTGTTGAAAGGGAAGTGCTTGAGATCAGTCTTAAGGTTTAGTGATCAGTCT
TCCTTTTGGTTGGTGCACTCACTATTTCTTAGGCCAACATCAGTTTTCGCGGCAGG
ATAATGACATTGGAATGTGGCTCTTTCGGGAGTGTTATAGCCTTTGTTGATACTGC
CTGCTTAGACTGAGGACCGCGCTTTTGCTAGGATGTTGGCATAATGATCTTAAG

77. ***Lipomyces tetrasporus*** **SWU-NGP 5-4-1**

> SWU-NGP 5-4-1 (544 bp)
GCCTTAGTAACTGCGAGTGAAGCGGCAAGAGCTCAAATTTGAAATCTGGCACCTT
CGGTGTCCGAGTTGTAATTTGTAGAAGCATCTTTTGGATTGGTCCTTGCATATGTTC
CTTGGAACAGGACATCATAGAGGGTGAGAATCCCGTTCATGGTGGGGAATCCAA
TTCTTTGTAAAGTGCTTTCGAAGAGTCGAGTTGTTTGGGAATGCAGCTCTAAGTGG
GTGGTAAATTCCATCTAAAGCTAAATATTGGCGAGAGACCGATAGCGAACAAGT
ACAGTGATGGAAAGATGAAAAGAACTCCGAAAGGAGAGTGAAAAAGGACGTGA
AATTGTTGAAAGGGAAGTGCTTGAGATCAGTCTTAAGGTTTAGTGATCAGTCTTCC
TTTTGGTTGGTGCACTCACTATTTCTTAGGCCAACATCAGTTTTCGCGGCAGGATA
ATGACATTGGAATGTGGCTCTTTCGGGAGTGTTATAGCCTTTGTTGATACTGCCTG
CTTAGACTGAGGACCGCGCTTTTGCTAGGATGTTGGCATAATGATCT

78. ***Lipomyces starkeyi*** **SWU-NATP 4-3**

>SWU-NATP 4-3 (556 bp)

AACAGGGATTGCCTTAGTAACTGCGAGTGAAGCGGCAAGAGCTCAAATTTGAAA
TCTGGCACCTTCGGTGTCCGAGTTGTAATTTGTAGAAGCATCTTTTGGATTGGTCCT
TGCATATGTTCCTTGGAACAGGACATCATAGAGGGTGAGAATCCCGTTCATGGTG
GGGAATCCAATTCTTTGTAAAGTGCTTTCGAAGAGTCGAGTTGTTTGGGAATGCA
GCTCTAAGTGGGTGGTAAATTCCATCTAAAGCTAAATATTGGCGAGAGACCGATA
GCGAACAAGTACAGTGATGGAAAGATGAAAAGAACTCCGAAAGGAGAGTGAAA
AAGGACGTGAAATTGTTGAAAGGGAAGTGCTTGAGATCAGTCTTAAGGTTTAGTG
ATCAGTCTTCCTTTTGGTTGGTGCACTCACTATTTCTTAGGCCAACATCAGTTTTGG
CGGCAGGATAATGACATTGGAATGTGGCTCTTTCGGGAGTGTTATAGCCTTTGTTG
ATACTGCCTGCTTAGACTGAGGACTGCGCTTTTGCTAGGATGTTGGCATAATGATC
TTA

79. ***Lipomyces starkeyi*** **SWU-NAP 14-4**

>SWU-NAP 14-4 (547 bp)

CCTTAGTAACTGCGAGTGAAGCGGCAAGAGCTCAAATTTGAAATCTGGCACCTTC
GGTGTCCGAGTTGTAATTTGTAGAAGCATCTTTTGGATTGGTCCTTGCATATGTTCC
TTGGAACAGGACATCATAGAGGGTGAGAATCCCGTTCATGGTGGGGAATCCAAT
TCTTTGTAAAGTGCTTTCGAAGAGTCGAGTTGTTTGGGAATGCAGCTCTAAGTGGG
TGGTAAATTCCATCTAAAGCTAAATATTGGCGAGAGACCGATAGCGAACAAGTA
CAGTGATGGAAAGATGAAAAGAACTCCGAAAGGAGAGTGAAAAAGGACGTGAA
ATTGTTGAAAGGGAAGTGCTTGAGATCAGTCTTAAGGTTTAGTGATCAGTCTTCCT
TTTGGTTGGTGCACTCACTATTTCTTAGGCCAACATCAGTTTTGGCGGCAGGATAA
TGACATTGGAATGTGGCTCTTTCGGGAGTGTTATAGCCTTTGTTGATACTGCCTGC
TTAGACAGAGGACTGCGCTTTTGCTAGGATGTTGGCATAATGATCTTAAG

80. ***Lipomyces tetrasporus*** **SWU-NGP 2-5**

>SWU-NGP 2-5 (550 bp)

GCCTTAGTAACTGCGAGTGAAGCGGCAAGAGCTCAAATTTGAAATCTGGCACCTT
CGGTGTCCGAGTTGTAATTTGTAGAAGCATCTTTTGGATTGGTCCTTGCATATGTTC
CTTGGAACAGGACATCATAGAGGGTGAGAATCCCGTTCATGGTGGGGAATCCAA
TTCTTTGTAAAGTGCTTTCGAAGAGTCGAGTTGTTTGGGAATGCAGCTCTAAGTGG
GTGGTAAATTCCATCTAAAGCTAAATATTGGCGAGAGACCGATAGCGAACAAGT

ACAGTGATGGAAAGATGAAAAGAACTCCGAAAGGAGAGTGAAAAAGGACGTGA
AATTGTTGAAAGGGAAGTGCTTGAGATCAGTCTTAAGGTTTAGTGATCAGTCTTCC
TTTTGGTTGGTGCACTCACTATTTCTTAGGCCAACATCAGTTTTCGCGGCAGGATA
ATGACATTGGAATGTGGCTCTTTCGGGAGTGTTATAGCCTTTGTTGATACTGCCTG
CTTAGACTGAGGACCGCGCTTTTGCTAGGATGTTGGCATAATGATCTTAAGCA

81. ***Lipomyces tetrasporus*** **SWU-NGP 2-6-2**
>SWU-NGP 2-6-2 (560 bp)
CAGGGATTGCCTTAGTAACTGCGAGTGAAGCGGCAAGAGCTCAAATTTGAAATCT
GGCACCTTCGGTGTCCGAGTTGTAATTTGTAGAAGCATCTTTTGGATTGGTCCTTG
CATATGTTCCTTGGAACAGGACATCATAGAGGGTGAGAATCCCGTTCATGGTGGG
GAATCCAATTCTTTGTAAAGTGCTTTCGAAGAGTCGAGTTGTTTGGGAATGCAGCT
CTAAGTGGGTGGTAAATTCCATCTAAAGCTAAATATTGGCGAGAGACCGATAGC
GAACAAGTACAGTGATGGAAAGATGAAAAGAACTCCGAAAGGAGAGTGAAAAA
GGACGTGAAATTGTTGAAAGGGAAGTGCTTGAGATCAGTCTTAAGGTTTAGTGAT
CAGTCTTCCTTTTGGTTGGTGCACTCACTATTTCTTAGGCCAACATCAGTTTTCGCG
GCAGGATAATGACATTGGAATGTGGCTCTTTCGGGAGTGTTATAGCCTTTGTTGAT
ACTGCCTGCTTAGACTGAGGACCGCGCTTTTGCTAGGATGTTGGCATAATGATCTT
AAGCAGC

82. ***Lipomyces starkeyi*** **SWU-NGP 5-3-3**
>SWU-NGP 5-3-3 (499 bp)
GGCACCTTCGGTGTCCGAGTTGTAATTTGTAGAAGCATCTTTTGGATTGGTCCTTG
CATATGTTCCTTGGAACAGGACATCATAGAGGGTGAGAATCCCGTTCATGGTGGG
GAATCCAATTCTTTGTAAAGTGCTTTCGAAGAGTCGAGTTGTTTGGGAATGCAGCT
CTAAGTGGGTGGTAAATTCCATCTAAAGCTAAATATTGGCGAGAGACCGATAGC
GAACAAGTACAGTGATGGAAAGATGAAAAGAACTCCGAAAGGAGAGTGAAAAA
GGACGTGAAATTGTTGAAAGGGAAGTGCTTGAGATCAGTCTTAAGGTTTAGTGAT
CAGTCTTCCTTTTGGTTGGTGCACTCACTATTTCTTAGGCCAACATCAGTTTTGGCG
GCAGGATAATGACATTGGAATGTGGCTCTTTCGGGAGTGTTATAGCCTTTGTTGAT
ACTGCCTGCTTAGACTGAGGACTGCGCTTTTGCTAGGATGTTGGCATAATGATCTT
A

83. *Lipomyces starkeyi* SWU-NGP 5-10-8

>SWU-NGP 5-10-8 (552 bp)

AGGGATTGCCTTAGTAACTGCGAGTGAAGCGGCAAGAGCTCAAATTTGAAATCT
GGCACCTTCGGTGTCCGAGTTGTAATTTGTAGAAGCATCTTTTGGATTGGTCCTTG
CATATGTTCCTTGGAACAGGACATCATAGAGGGTGAGAATCCCGTTCATGGTGGG
GAATCCAATTCTTTGTAAAGTGCTTTCGAAGAGTCGAGTTGTTTGGGAATGCAGCT
CTAAGTGGGTGGTAAATTCCATCTAAAGCTAAATATTGGCGAGAGACCGATAGC
GAACAAGTACAGTGATGGAAAGATGAAAAGAACTCCGAAAGGAGAGTGAAAAA
GGACGTGAAATTGTTGAAAGGGAAGTGCTTGAGATCAGTCTTAAGGTTTAGTGAT
CAGTCTTCCTTTTGGTTGGTGCACTCACTATTTCTTAGGCCAACATCAGTTTTGGCG
GCAGGATAATGGCATTGGAATGTGGCTCTTTCGGGAGTGTTATAGCCTTTGTTGAT
ACTGCCTGCTTAGACTGAGGACTGCGCTTTTGCTAGGATGTTGGCATAATGATCTT

84. *Lipomyces tetrasporus* SWU-NGTP 4-2

>SWU-NGTP 4-2 (538 bp)

ATTGCCTTAGTAACTGCGAGTGAAGCGGCAAGAGCTCAAATTTGAAATCTGGCAC
CTTCGGTGTCCGAGTTGTAATTTGTAGAAGCATCTTTTGGATTGGTCCTTGCATATG
TTCCTTGGAACAGGACATCATAGAGGGTGAGAATCCCGTTCATGGTGGGGAATCC
AATTCTTTGTAAAGTGCTTTCGAAGAGTCGAGTTGTTTGGGAATGCAGCTCTAAGT
GGGTGGTAAATTCCATCTAAAGCTAAATATTGGCGAGAGACCGATAGCGAACAA
GTACAGTGATGGAAAGATGAAAAGAACTCCGAAAGGAGAGTGAAAAAGGACGT
GAAATTGTTGAAAGGGAAGTGCTTGAGATCAGTCTTAAGGTTTAGTGATCAGTCT
TCCTTTTGGTTGGTGCACTCACTATTTCTTAGGCCAACATCAGTTTTCGCGGCAGG
ATAATGACATTGGAATGTGGCTCTTTCGGGAGTGTTATAGCCTTTGTTGATACTGC
CTGCTTAGACTGAGGACCGCGCTTTTGCTAGGATGTTGGCA

85. *Lipomyces starkeyi* SWU-NGP 14-3-1

>SWU-NGP 14-3-1 (498 bp)

CACCTTCGGTGTCCGAGTTGTAATTTGTAGAAGCATCTTTTGGATTGGTCCTTGCAT
ATGTTCCTTGGAACAGGACATCATAGAGGGTGAGAATCCCGTTCATGGTGGGGA
ATCCAATTCTTTGTAAAGTGCTTTCGAAGAGTCGAGTTGTTTGGGAATGCAGCTCT
AAGTGGGTGGTAAATTCCATCTAAAGCTAAATATTGGCGAGAGACCGATAGCGA
ACAAGTACAGTGATGGAAAGATGAAAAGAACTCCGAAAGGAGAGTGAAAAAGG

ACGTGAAATTGTTGAAAGGGAAGTGCTTGAGATCAGTCTTAAGGTTTAGTGATCA
GTCTTCCTTTTGGTTGGTGCACTCACTATTTCTTAGGCCAACATCAGTTTTGGCGGC
AGGATAATGACATTGGAATGTGGCTCTTTCGGGACTGTTATAGCCTTTGTTGATAC
TGCCAGCTGATACAGACGACGGCGCTTTTGCTAGGATGTTGGCATAATGATCTTA
A

86. ***Rhodotorula mucilaginosa*** **SWU-NGP 14-3-3**

>SWU-NGP 14-3-3 (500 bp)
GCACCTTCGGTGTCCGAGTTGTAATCTCTAGAAATGTTTTCCGCGTTGGACCGCAC
ACAAGTCTGTTGGAATACAGCGGCATAGTGGTGAGACCCCCGTATATGGTGCGG
ACGCCCAGCGCTTTGTGATACATTTTCGAAGAGTCGAGTTGTTTGGGAATGCAGC
TCAAATTGGGTGGTAAATTCCATCTAAAGCTAAATATTGGCGAGAGACCGATAGC
GAACAAGTACCGTGAGGGAAAGATGAAAAGCACTTTGGAAAGAGAGTTAACAG
TACGTGAAATTGTTGGAAGGGAAACGCTTGAAGTCAGACTTGCTTGCTGAGCAAT
CGGTTTGCAGGCCAGCATCAGTTTTCCGGGATGGATAATGGTAGAGAGAAGGTA
GCAGTTTCGGCTGTGTTATAGCTCTCTGCTGGATACATCTTGGGGGACTGAGGAAC
GCAGTGTGCCTTTGGCGGGGGTTTCGACCTCTTCACACTTAGGATGCTGGTGGAAT
GGCTTT

87. ***Lipomyces starkeyi*** **SWU-NGP 1-5-4**

>SWU-NGP 1-5-4 (554 bp)
AGGGATTGCCTTAGTAACTGCGAGTGAAGCGGCAAGAGCTCAAATTTGAAATCT
GGCACCTTCGGTGTCCGAGTTGTAATTTGTAGAAGCATCTTTTGGATTGGTCCTTG
CATATGTTCCTTGGAACAGGACATCATAGAGGGTGAGAATCCCGTTCATGGTGGG
GAATCCAATTCTTTGTAAAGTGCTTTCGAAGAGTCGAGTTGTTTGGGAATGCAGCT
CTAAGTGGGTGGTAAATTCCATCTAAAGCTAAATATTGGCGAGAGACCGATAGC
GAACAAGTACAGTGATGGAAAGATGAAAAGAACTCCGAAAGGAGAGTGAAAAA
GGACGTGAAATTGTTGAAAGGGAAGTGCTTGAGATCAGTCTTAAGGTTTAGTGAT
CAGTCTTCCTTTTGGTTGGTGCACTCACTATTTCTTAGGCCAACATCAGTTTTGGCG
GCAGGATAATGACATTGGAATGTGGCTCTTTCGGGAGTGTTATAGCCTTTGTTGAT
ACTGCCAGCTGATACAGACGACGGCGCTTTTGCTAGGATGTTGGCATAATGATCT
TAA

88. ***Lipomyces starkeyi*** **SWU-NGTP 4-12-1**

>SWU-NGTP 4-12-1 (498 bp)

CACCTTCGGTGTCCGAGTTGTAATTTGTAGAAGCATCTTTTGGATTGGTCCTTGCAT
ATGTTCCTTGGAACAGGACATCACTGAGGGTGAGAATCCCGTTCATGGTGGGGAA
TCCAATTCTTTGTAAAGTGCTTTCGAAGAGTCGAGTTGTTTGGGAATGCAGCTCTA
AGTGGGTGGTAAATTCCATCTAAAGCTAAATATTGGCGAGAGACCGATAGCGAA
CAAGTACAGTGATGGAAAGATGAAAAGAACTCCGAAAGGAGAGTGAAAAAGGA
CGTGAAATTGTTGAAAGGGAAGTGCTTGAGATCAGTCTTAAGGTTTAGTGATCAG
TCTTCCTTTTGGTTGGTGCACTCACTATTTCTTAGGCCAACATCAGTTTTGGCGGCA
GGATAATGACATTGGAATGTGGCTCTTTCGGGAGTGTTATAGCCTTTGTTGATACT
GCCAGCTGATACAGACGACGGCGCTTTTGCTAGGATGTTGGCATAATGATCTTAA

89. ***Saitozyma podzolica*** **SWU-NGP 5-4-2**

>SWU-NGP 5-4-2 (570 bp)

TTCCCTTAGTAGCGGCGAGCGAAGCGGGAAGAGCTCAAATTTGAAAGCTGGCGT
CCTACGGGCGTCCGCATTGTAATCTATAGAGGCGTTTTCTGTGCTGGACCGTGTCC
AAGTCCCTTGGAACAGGGTATCAAAGAGGGTGACAATCCCGTACTTGACACGAC
AACCAGTGCTCTGTGATACGTCTTCTACGAGTCGAGTTGTTTGGGAATGCAGCTCA
AAATGGGTGGTGAATTCCATCTAAAGCTAAATATTGGCGAGAGACCGATAGCGA
ACAAGTACCGTGAGGGAAAGATGAAAAGCACTTTGGAAAGAGAGTTAAACAGT
ATGTGAAATTGTTGAAAGGGAAACGATGGAGGTCAGTCGTGTCTGTGGGATTCAG
CCGTCTCTGGCGGTGTACTTCCCACAGACGGGTCAACATCAGTTTTGGCTGGCGG
ATAAAGGCAGGAGGAAGGTGGCACCCCCGGGTGTGTTATAGCCTCTTGTTGCATA
CGCTGGCTGAGACTGAGGAATGCAGCTCGCCTTTATGGCCGGGGTTCGCCCACGT
TCGAGCTTAGGATGTTGACATAA

90. ***Lipomyces tetrasporus*** **SWU-NGP 5-6-1**

>SWU-NGP 5-6-1 (545 bp)

CCTTAGTAACTGCGAGTGAAGCGGCAAGAGCTCAAATTTGAAATCTGGCACCTTC
GGTGTCCGAGTTGTAATTTGTAGAAGCATCTTTTGGATTGGTCCTTGCATATGTTCC
TTGGAACAGGACATCATAGAGGGTGAGAATCCCGTTCATGGTGGGGAATCCAAT
TCTTTGTAAAGTGCTTTCGAAGAGTCGAGTTGTTTGGGAATGCAGCTCTAAGTGGG
TGGTAAATTCCATCTAAAGCTAAATATTGGCGAGAGACCGATAGCGAACAAGTA
CAGTGATGGAAAGATGAAAAGAACTCCGAAAGGAGAGTGAAAAAGGACGTGAA

ATTGTTGAAAGGGAAGTGCTTGAGATCAGTCTTAAGGTTTAGTGATCAGTCTTCCT
TTTGGTTGGTGCACTCACTATTTCTTAGGCCAACATCAGTTTTCGCGGCAGGATAA
TGACATTGGAATGTGGCTCTTTCGGGAGTGTTATAGCCTTTGTTGATACTGCCTGC
TTAGACTGAGGACCGCGCTTTTGCTAGGATGTTGGCATAATGATCTTA

91. ***Lipomyces tetrasporus*** **SWU-NGP 1-1**

>SWU-NGP 1-1 (500 bp)

GCCTTAGTAACTGCGAGTGAAGCGGCAAGAGCTCAAATTTGAAATCTGGCACCTT
CGGTGTCCGAGTTGTAATTTGTAGAAGCATCTTTTGGATTGGTCCTTGCATATGTTC
CTTGGAACAGGACATCATAGAGGGTGAGAATCCCGTTCATGGTGGGGAATCCAA
TTCTTTGTAAAGTGCTTTCGAAGAGTCGAGTTGTTTGGGAATGCAGCTCTAAGTGG
GTGGTAAATTCCATCTAAAGCTAAATATTGGCGAGAGACCGATAGCGAACAAGT
ACAGTGATGGAAAGATGAAAAGAACTCCGAAAGGAGAGTGAAAAAGGACGTGA
AATTGTTGAAAGGGAAGTGCTTGAGATCAGTCTTAAGGTTTAGTGATCAGTCTTCC
TTTTGGTTGGTGCACTCACTATTTCTTAGGCCAACATCAGTTTTCGCGGCAGGATA
ATGACATTGGAATGTGGCTCTTTCGGGAGTGTTATAGCCTTTGTTGATACTGCCTG
CTT

92. ***Lipomyces tetrasporus*** **SWU-NGP 3-2**

>SWU-NGP 3-2 (459 bp)

TTGGATTGGTCCTTGCATATGTTCCTTGGAACAGGACATCATAGAGGGTGAGAAT
CCCGTTCATGGTGGGGAATCCAATTCTTTGTAAAGTGCTTTCGAAGAGTCGAGTTG
TTTGGGAATGCAGCTCTAAGTGGGTGGTAAATTCCATCTAAAGCTAAATATTGGC
GAGAGACCGATAGCGAACAAGTACAGTGATGGAAAGATGAAAAGAACTCCGAA
AGGAGAGTGAAAAAGGACGTGAAATTGTTGAAAGGGAAGTGCTTGAGATCAGTC
TTAAGGTTTAGTGATCAGTCTTCCTTTTGGTTGGTGCACTCACTATTTCTTAGGCCA
ACATCAGTTTTCGCGGCAGGATAATGACATTGGAATGTGGCTTTTTCCGGGAGTGT
TATAGCCTTTGTTGATACTGCCTGCTTAGACTGAGGACCGCGCTTTTGCTAGGATG
TTGGCATAATGATCTTA

93. ***Lipomyces tetrasporus*** **SWU-NGP 3-1**

>SWU-NGP 3-1 (551 bp)

GGATTGCCTTAGTAACTGCGAGTGAAGCGGCAAGAGCTCAAATTTGAAATCTGGC
ACCTTCGGTGTCCGAGTTGTAATTTGTAGAAGCATCTTTTGGATTGGTCCTTGCAT

ATGTTCCTTGGAACAGGACATCATAGAGGGTGAGAATCCCGTTCATGGTGGGGA
ATCCAATTCTTTGTAAAGTGCTTTCGAAGAGTCGAGTTGTTTGGGAATGCAGCTCT
AAGTGGGTGGTAAATTCCATCTAAAGCTAAATATTGGCGAGAGACCGATAGCGA
ACAAGTACAGTGATGGAAAGATGAAAAGAACTCCGAAAGGAGAGTGAAAAAGG
ACGTGAAATTGTTGAAAGGGAAGTGCTTGAGATCAGTCTTAAGGTTTAGTGATCA
GTCTTCCTTTTGGTTGGTGCACTCACTATTTCTTAGGCCAACATCAGTTTTCGCGGC
AGGATAATGACATTGGAATGTGGCTCTTTCGGGAGTGTTATAGCCTTTGTTGATAC
TGCCTGCTTAGACTGAGGACCGCGCTTTTGCTAGGATGTTGGCATAATGATCTTA

94. ***Lipomyces tetrasporus*** **SWU-NGP 6-3**
>SWU-NGP 6-3 (559 bp)
AACAGGGATTGCCTTAGTAACTGCGAGTGAAGCGGCAAGAGCTCAAATTTGAAA
TCTGGCACCTTCGGTGTCCGAGTTGTAATTTGTAGAAGCATCTTTTGGATTGGTCCT
TGCATATGTTCCTTGGAACAGGACATCATAGAGGGTGAGAATCCCGTTCATGGTG
GGGAATCCAATTCTTTGTAAAGTGCTTTCGAAGAGTCGAGTTGTTTGGGAATGCA
GCTCTAAGTGGGTGGTAAATTCCATCTAAAGCTAAATATTGGCGAGAGACCGATA
GCGAACAAGTACAGTGATGGAAAGATGAAAAGAACTCCGAAAGGAGAGTGAAA
AAGGACGTGAAATTGTTGAAAGGGAAGTGCTTGAGATCAGTCTTAAGGTTTAGTG
ATCAGTCTTCCTTTTGGTTGGTGCACTCACTATTTCTTAGGCCAACATCAGTTTTCG
CGGCAGGATAATGACATTGGAATGTGGCTCTTTCGGGAGTGTTATAGCCTTTGTTG
ATACTGCCTGCTTAGACTGAGGACCGCGCTTTTGCTAGGATGTTGGCATAATGATC
TTAAGC

95. ***Lipomyces tetrasporus*** **SWU-NGP 2-1-1**
>SWU-NGP 2-1-1 (463 bp)
TCTTTTGGATTGGTCCTTGCATATGTTCCTTGGAACAGGACATCATAGAGGGTGAG
AATCCCGTTCATGGTGGGGAATCCAATTCTTTGTAAAGTGCTTTCGAAGAGTCGA
GTTGTTTGGGAATGCAGCTCTAAGTGGGTGGTAAATTCCATCTAAAGCTAAATATT
GGCGAGAGACCGATAGCGAACAAGTACAGTGATGGAAAGATGAAAAGAACTCC
GAAAGGAGAGTGAAAAAGGACGTGAAATTGTTGAAAGGGAAGTGCTTGAGATC
AGTCTTAAGGTTTAGTGATCAGTCTTCCTTTTGGTTGGTGCACTCACTATTTCTTAG
GCCAACATCAGTTTTCGCGGCAGGATAATGACATTGGAATGTGGCTCTTTCGGGA
GTGTTATAGCCTTTGTTGATACTGCCTGCTTAGACTGAGGACCGCGCTTTTGCTAG
GATGTTGGCATAATGATCTTAA

96. ***Lipomyces starkeyi*** **SWU-NGTP 4-12-3**

>SWU-NGTP 4-12-3 (450 bp)

GGTCCTTGCATATGTTCCTTGGAACAGGACATCATAGAGGGTGAGAATCCCGTTC
ATGGTGGGGAATCCAATTCTTTGTAAAGTGCTTTCGAAGAGTCGAGTTGTTTGGGA
ATGCAGCTCTAAGTGGGTGGTAAATTCCATCTAAAGCTAAATATTGGCGAGAGAC
CGATAGCGAACAAGTACAGTGATGGAAAGATGAAAAGAACTCCGAAAGGAGAG
TGAAAAAGGACGTGAAATTGTTGAAAGGGAAGTGCTTGAGATCAGTCTTAAGGT
TTAGTGATCAGTCTTCCTTTTGGTTGGTGCACTCACTATTTCTTAGGCCAACATCAG
TTTTGGCGGCAGGATAATGACATTGGAATGTGGCTCTTTCGGGAGTGTTATAGCCT
TTGTTGATACTGCCTGCTTAGACAGAGGACTGCGCTTTTGCTAGGATGTTGGCATA
ATGATCTT

97. ***Lipomyces tetrasporu*****s SWU-NGP 4-4**

>SWU-NGP 4-4 (554 bp)

TTGCCTTAGTAACTGCGAGTGAAGCGGCAAGAGCTCAAATTTGAAATCTGGCACC
TTCGGTGTCCGAGTTGTAATTTGTAGAAGCATCTTTTGGATTGGTCCTTGCATATGT
TCCTTGGAACAGGACATCATAGAGGGTGAGAATCCCGTTCATGGTGGGGAATCC
AATTCTTTGTAAAGTGCTTTCGAAGAGTCGAGTTGTTTGGGAATGCAGCTCTAAGT
GGGTGGTAAATTCCATCTAAAGCTAAATATTGGCGAGAGACCGATAGCGAACAA
GTACAGTGATGGAAAGATGAAAAGAACTCCGAAAGGAGAGTGAAAAAGGACGT
GAAATTGTTGAAAGGGAAGTGCTTGAGATCAGTCTTAAGGTTTAGTGATCAGTCT
TCCTTTTGGTTGGTGCACTCACTATTTCTTAGGCCAACATCAGTTTTCGCGGCAGG
ATAATGACATTGGAATGTGGCTCTTTCGGGAGTGTTATAGCCTTTGTTGATACTGC
CTGCTTAGACTGAGGACCGCGCTTTTGCTAGGATGTTGGCATAATGATCTTAAGC
AGC

98. ***Lipomyces starkeyi*** **SWU-NGP 6-6**

>SWU-NGP 6-6 (560 bp)

CAGGGATTGCCTTAGTAACTGCGAGTGAAGCGGCAAGAGCTCAAATTTGAAATCT
GGCACCTTCGGTGTCCGAGTTGTAATTTGTAGAAGCATCTTTTGGATTGGTCCTTG
CATATGTTCCTTGGAACAGGACATCATAGAGGGTGAGAATCCCGTTCATGGTGGG
GAATCCAATTCTTTGTAAAGTGCTTTCGAAGAGTCGAGTTGTTTGGGAATGCAGCT
CTAAGTGGGTGGTAAATTCCATCTAAAGCTAAATATTGGCGAGAGACCGATAGC

GAACAAGTACAGTGATGGAAAGATGAAAAGAACTCCGAAAGGAGAGTGAAAAA
GGACGTGAAATTGTTGAAAGGGAAGTGCTTGAGATCAGTCTTAAGGTTTAGTGAT
CAGTCTTCCTTTTGGTTGGTGCACTCACTATTTCTTAGGCCAACATCAGTTTTGGCG
GCAGGATAATGACATTGGAATGTGGCTCTTTCGGGAGTGTTATAGCCTTTGTTGAT
ACTGCCTGCTTAGACTGAGGACTGCGCTTTTGCTAGGATGTTGGCATAATGATCTT
AAGCAGC

99. ***Lipomyces tetrasporus*** **SWU-NAP 4-2-2**

>SWU-NAP 4-2-2 (554 bp)
GGATTGCCTTAGTAACTGCGAGTGAAGCGGCAAGAGCTCAAATTTGAAATCTGGC
ACCTTCGGTGTCCGAGTTGTAATTTGTAGAAGCATCTTTTGGATTGGTCCTTGCAT
ATGTTCCTTGGAACAGGACATCATAGAGGGTGAGAATCCCGTTCATGGTGGGGA
ATCCAATTCTTTGTAAAGTGCTTTCGAAGAGTCGAGTTGTTTGGGAATGCAGCTCT
AAGTGGGTGGTAAATTCCATCTAAAGCTAAATATTGGCGAGAGACCGATAGCGA
ACAAGTACAGTGATGGAAAGATGAAAAGAACTCCGAAAGGAGAGTGAAAAAGG
ACGTGAAATTGTTGAAAGGGAAGTGCTTGAGATCAGTCTTAAGGTTTAGTGATCA
GTCTTCCTTTTGGTTGGTGCACTCACTATTTCTTAGGCCAACATCAGTTTTCGCGGC
AGGATAATGACATTGGAATGTGGCTCTTTCGGGAGTGTTATAGCCTTTGTTGATAC
TGCCTGCTTAGACTGAGGACCGCGCTTTTGCTAGGATGTTGGCATAATGATCTTAA
GC

100. ***Lipomyces tetrasporus*** **SWU-NAP 5-4**

>SWU-NAP 5-4 (555 bp)
AGGGATTGCCTTAGTAACTGCGAGTGAAGCGGCAAGAGCTCAAATTTGAAATCT
GGCACCTTCGGTGTCCGAGTTGTAATTTGTAGAAGCATCTTTTGGATTGGTCCTTG
CATATGTTCCTTGGAACAGGACATCATAGAGGGTGAGAATCCCGTTCATGGTGGG
GAATCCAATTCTTTGTAAAGTGCTTTCGAAGAGTCGAGTTGTTTGGGAATGCAGCT
CTAAGTGGGTGGTAAATTCCATCTAAAGCTAAATATTGGCGAGAGACCGATAGC
GAACAAGTACAGTGATGGAAAGATGAAAAGAACTCCGAAAGGAGAGTGAAAAA
GGACGTGAAATTGTTGAAAGGGAAGTGCTTGAGATCAGTCTTAAGGTTTAGTGAT
CAGTCTTCCTTTTGGTTGGTGCACTCACTATTTCTTAGGCCAACATCAGTTTTCGCG
GCAGGATAATGACATTGGAATGTGGCTCTTTCGGGAGTGTTATAGCCTTTGTTGAT
ACTGCCTGCTTAGACTGAGGACCGCGCTTTTGCTAGGATGTTGGCATAATGATCTT
AAG

101. ***Lipomyces starkeyi*** **SWU-NGP 14-1**

>SWU-NGP 14-1 (504 bp)

GCCTTAGTAACTGCGAGTGAAGCGGCAAGAGCTCAAATTTGAAATCTGGCACCTT
CGGTGTCCGAGTTGTAATTTGTAGAAGCATCTTTTGGATTGGTCCTTGCATATGTTC
CTTGGAACAGGACATCATAGAGGGTGAGAATCCCGTTCATGGTGGGGAATCCAA
TTCTTTGTAAAGTGCTTTCGAAGAGTCGAGTTGTTTGGGAATGCAGCTCTAAGTGG
GTGGTAAATTCCATCTAAAGCTAAATATTGGCGAGAGACCGATAGCGAACAAGT
ACAGTGATGGAAAGATGAAAAGAACTCCGAAAGGAGAGTGAAAAAGGACGTGA
AATTGTTGAAAGGGAAGTGCTTGAGATCAGTCTTAAGGTTTAGTGATCAGTCTTCC
TTTTGGTTGGTGCACTCACTATTTCTTAGGCCAACATCAGTTTTGGCGGCAGGATA
ATGACATTGGAATGTGGCTCTTTCGGGAGTGTTATAGCCTTTGTTGATACTGCCTG
CTTAGAC

102. ***Lipomyces starkeyi*** **SWU-NGP 14-9**

>SWU-NGP 14-9 (557 bp)

GGGATTGCCTTAGTAACTGCGAGTGAAGCGGCAAGAGCTCAAATTTGAAATCTG
GCACCTTCGGTGTCCGAGTTGTAATTTGTAGAAGCATCTTTTGGATTGGTCCTTGC
ATATGTTCCTTGGAACAGGACATCATAGAGGGTGAGAATCCCGTTCATGGTGGGG
AATCCAATTCTTTGTAAAGTGCTTTCGAAGAGTCGAGTTGTTTGGGAATGCAGCTC
TAAGTGGGTGGTAAATTCCATCTAAAGCTAAATATTGGCGAGAGACCGATAGCG
AACAAGTACAGTGATGGAAAGATGAAAAGAACTCCGAAAGGAGAGTGAAAAAG
GACGTGAAATTGTTGAAAGGGAAGTGCTTGAGATCAGTCTTAAGGTTTAGTGATC
AGTCTTCCTTTTGGTTGGTGCACTCACTATTTCTTAGGCCAACATCAGTTTTGGCGG
CAGGATAATGACATTGGAATGTGGCTCTTTCGGGAGTGTTATAGCCTTTGTTGATA
CTGCCTGCTTAGACTGAGGACTGCGCTTTTGCTAGGATGTTGGCATAATGATCTTA
AGCAG

103. ***Lipomyces tetrasporus*** **SWU-NGP 5-9-2**

>SWU-NGP 5-9-2 (541 bp)

CTTAGTAACTGCGAGTGAAGCGGCAAGAGCTCAAATTTGAAATCTGGCACCTTCG
GTGTCCGAGTTGTAATTTGTAGAAGCATCTTTTGGATTGGTCCTTGCATATGTTCCT
TGGAACAGGACATCATAGAGGGTGAGAATCCCGTTCATGGTGGGGAATCCAATT
CTTTGTAAAGTGCTTTCGAAGAGTCGAGTTGTTTGGGAATGCAGCTCTAAGTGGGT

GGTAAATTCCATCTAAAGCTAAATATTGGCGAGAGACCGATAGCGAACAAGTAC
AGTGATGGAAAGATGAAAAGAACTCCGAAAGGAGAGTGAAAAAGGACGTGAAA
TTGTTGAAAGGGAAGTGCTTGAGATCAGTCTTAAGGTTTAGTGATCAGTCTTCCTT
TTGGTTGGTGCACTCACTATTTCTTAGGCCAACATCAGTTTTCGCGGCAGGATAAT
GACATTGGAATGTGGCTCTTTCGGGAGTGTTATAGCCTTTGTTGATACTGCCTGCT
TAGACTGAGGACCGCGCTTTTGCTAGGATGTTGGCATAATGATC

104. ***Lipomyces starkeyi*** **SWU-NGTP 5-8**

>SWU-NGTP 5-8 (544 bp)
ATTGCCTTAGTAACTGCGAGTGAAGCGGCAAGAGCTCAAATTTGAAATCTGGCAC
CTTCGGTGTCCGAGTTGTAATTTGTAGAAGCATCTTTTGGATTGGTCCTTGCATATG
TTCCTTGGAACAGGACATCATAGAGGGTGAGAATCCCGTTCATGGTGGGGAATCC
AATTCTTTGTAAAGTGCTTTCGAAGAGTCGAGTTGTTTGGGAATGCAGCTCTAAGT
GGGTGGTAAATTCCATCTAAAGCTAAATATTGGCGAGAGACCGATAGCGAACAA
GTACAGTGATGGAAAGATGAAAAGAACTCCGAAAGGAGAGTGAAAAAGGACGT
GAAATTGTTGAAAGGGAAGTGCTTGAGATCAGTCTTAAGGTTTAGTGATCAGTCT
TCCTTTTGGTTGGTGCACTCACTATTTCTTAGGCCAACATCAGTTTTGGCGGCAGG
ATAATGACATTGGAATGTGGCTCTTTCGGGAGTGTTATAGCCTTTGTTGATACTGC
CTGCTTAGACTGAGGACTGCGCTTTTGCTAGGATGTTGGCATAATGA

105. ***Lipomyces tetrasporus*** **SWU-NGP 1-4**

>SWU-NGP 1-4 (551 bp)
CCTTAGTAACTGCGAGTGAAGCGGCAAGAGCTCAAATTTGAAATCTGGCACCTTC
GGTGTCCGAGTTGTAATTTGTAGAAGCATCTTTTGGATTGGTCCTTGCATATGTTCC
TTGGAACAGGACATCATAGAGGGTGAGAATCCCGTTCATGGTGGGGAATCCAAT
TCTTTGTAAAGTGCTTTCGAAGAGTCGAGTTGTTTGGGAATGCAGCTCTAAGTGGG
TGGTAAATTCCATCTAAAGCTAAATATTGGCGAGAGACCGATAGCGAACAAGTA
CAGTGATGGAAAGATGAAAAGAACTCCGAAAGGAGAGTGAAAAAGGACGTGAA
ATTGTTGAAAGGGAAGTGCTTGAGATCAGTCTTAAGGTTTAGTGATCAGTCTTCCT
TTTGGTTGGTGCACTCACTATTTCTTAGGCCAACATCAGTTTTCGCGGCAGGATAA
TGACATTGGAATGTGGCTCTTTCGGGAGTGTTATAGCCTTTGTTGATACTGCCTGC
TTAGACTGAGGACCGCGCTTTTGCTAGGATGTTGGCATAATGATCTTAAGCAGC

106. ***Lipomyces starkeyi*** **SWU-NGTP 4-6**

>SWU-NGTP 4-6 (544 bp)

GTAACTGCGAGTGAAGCGGCAAGAGCTCAAATTTGAAATCTGGCACCTTCGGTGT
CCGAGTTGTAATTTGTAGAAGCATCTTTTGGATTGGTCCTTGCATATGTTCCTTGGA
ACAGGACATCATAGAGGGTGAGAATCCCGTTCATGGTGGGGAATCCAATTCTTTG
TAAAGTGCTTTCGAAGAGTCGAGTTGTTTGGGAATGCAGCTCTAAGTGGGTGGTA
AATTCCATCTAAAGCTAAATATTGGCGAGAGACCGATAGCGAACAAGTACAGTG
ATGGAAAGATGAAAAGAACTCCGAAAGGAGAGTGAAAAAGGACGTGAAATTGT
TGAAAGGGAAGTGCTTGAGATCAGTCTTAAGGTTTAGTGATCAGTCTTCCTTTTGG
TTGGTGCACTCACTATTTCTTAGGCCAACATCAGTTTTGGCGGCAGGATAATGACA
TTGGAATGTGGCTCTTTCGGGAGTGTTATAGCCTTTGTTGATACTGCCTGCTTAGA
CTGAGGACTGCGCTTTTGCTAGGATGTTGGCATAATGATCTTAAGCA

107. ***Lipomyces tetrasporus*** **SWU-NGP 2-3**

>SWU-NGP 2-3 (524 bp)

GTAACTGCGAGTGAAGCGGCAAGAGCTCAAATTTGAAATCTGGCACCTTCGGTGT
CCGAGTTGTAATTTGTAGAAGCATCTTTTGGATTGGTCCTTGCATATGTTCCTTGGA
ACAGGACATCATAGAGGGTGAGAATCCCGTTCATGGTGGGGAATCCAATTCTTTG
TAAAGTGCTTTCGAAGAGTCGAGTTGTTTGGGAATGCAGCTCTAAGTGGGTGGTA
AATTCCATCTAAAGCTAAATATTGGCGAGAGACCGATAGCGAACAAGTACAGTG
ATGGAAAGATGAAAAGAACTCCGAAAGGAGAGTGAAAAAGGACGTGAAATTGT
TGAAAGGGAAGTGCTTGAGATCAGTCTTAAGGTTTAGTGATCAGTCTTCCTTTTGG
TTGGTGCACTCACTATTTCTTAGGCCAACATCAGTTTTCGCGGCAGGATAATGACA
TTGGAATGTGGCTCTTTCGGGAGTGTTATAGCCTTTGTTGATACTGCCTGCTTAGA
CTGAGGACCGCGCTTTTGCTAGGATGT

108. ***Lipomyces starkey*****i SWU-NGTP 4-11**

>SWU-NGTP 4-11 (534 bp)

GCCTTAGTAACTGCGAGTGAAGCGGCAAGAGCTCAAATTTGAAATCTGGCACCTT
CGGTGTCCGAGTTGTAATTTGTAGAAGCATCTTTTGGATTGGTCCTTGCATATGTTC
CTTGGAACAGGACATCATAGAGGGTGAGAATCCCGTTCATGGTGGGGAATCCAA
TTCTTTGTAAAGTGCTTTCGAAGAGTCGAGTTGTTTGGGAATGCAGCTCTAAGTGG
GTGGTAAATTCCATCTAAAGCTAAATATTGGCGAGAGACCGATAGCGAACAAGT
ACAGTGATGGAAAGATGAAAAGAACTCCGAAAGGAGAGTGAAAAAGGACGTGA

AATTGTTGAAAGGGAAGTGCTTGAGATCAGTCTTAAGGTTTAGTGATCAGTCTTCC
TTTTGGTTGGTGCACTCACTATTTCTTAGGCCAACATCAGTTTTGGCGGCAGGATA
ATGACATTGGAATGTGGCTCTTTCGGGAGTGTTATAGCCTTTGTTGATACTGCCTG
CTTAGACTGAGGACTGCGCTTTTGCTAGGATGTTGGC

109. ***Lipomyces tetrasporus*** **SWU-NGP 2-2**

>SWU-NGP 2-2 (540 bp)

GATTGCCTTAGTAACTGCGAGTGAAGCGGCAAGAGCTCAAATTTGAAATCTGGCA
CCTTCGGTGTCCGAGTTGTAATTTGTAGAAGCATCTTTTGGATTGGTCCTTGCATAT
GTTCCTTGGAACAGGACATCATAGAGGGTGAGAATCCCGTTCATGGTGGGGAATC
CAATTCTTTGTAAAGTGCTTTCGAAGAGTCGAGTTGTTTGGGAATGCAGCTCTAAG
TGGGTGGTAAATTCCATCTAAAGCTAAATATTGGCGAGAGACCGATAGCGAACA
AGTACAGTGATGGAAAGATGAAAAGAACTCCGAAAGGAGAGTGAAAAAGGACG
TGAAATTGTTGAAAGGGAAGTGCTTGAGATCAGTCTTAAGGTTTAGTGATCAGTC
TTCCTTTTGGTTGGTGCACTCACTATTTCTTAGGCCAACATCAGTTTTCGCGGCAGG
ATAATGACATTGGAATGTGGCTCTTTCGGGAGTGTTATAGCCTTTGTTGATACTGC
CTGCTTAGACTGAGGACCGCGCTTTTGCTAGGATGTTGGCAT

110. ***Lipomyces starkeyi*** **SWU-NGTP 4-5**

>SWU-NGTP 4-5 (532 bp)

GCCTTAGTAACTGCGAGTGAAGCGGCAAGAGCTCAAATTTGAAATCTGGCACCTT
CGGTGTCCGAGTTGTAATTTGTAGAAGCATCTTTTGGATTGGTCCTTGCATATGTTC
CTTGGAACAGGACATCATAGAGGGTGAGAATCCCGTTCATGGTGGGGAATCCAA
TTCTTTGTAAAGTGCTTTCGAAGAGTCGAGTTGTTTGGGAATGCAGCTCTAAGTGG
GTGGTAAATTCCATCTAAAGCTAAATATTGGCGAGAGACCGATAGCGAACAAGT
ACAGTGATGGAAAGATGAAAAGAACTCCGAAAGGAGAGTGAAAAAGGACGTGA
AATTGTTGAAAGGGAAGTGCTTGAGATCAGTCTTAAGGTTTAGTGATCAGTCTTCC
TTTTGGTTGGTGCACTCACTATTTCTTAGGCCAACATCAGTTTTGGCGGCAGGATA
ATGGCATTGGAATGTGGCTCTTTCGGGAGTGTTATAGCCTTTGTTGATACTGCCTG
CTTAGACTGAGGACTGCGCTTTTGCTAGGATGTTG

111. ***Lipomyces starkeyi*** **SWU-NAPH 3-6**

>SWU-NAPH 3-6 (518 bp)

ACTGCGAGTGAAGCGGCAAGAGCTCAAATTTGAAATCTGGCACCTTCGGTGTCCG
AGTTGTAATTTGTAGAAGCATCTTTTGGATTGGTCCTTGCATATGTTCCTTGGAAC
AGGACATCATAGAGGGTGAGAATCCCGTTCATGGTGGGGAATCCAATTCTTTGTA
AAGTGCTTTCGAAGAGTCGAGTTGTTTGGGAATGCAGCTCTAAGTGGGTGGTAAA
TTCCATCTAAAGCTAAATATTGGCGAGAGACCGATAGCGAACAAGTACAGTGAT
GGAAAGATGAAAAGAACTCCGAAAGGAGAGTGAAAAAGGACGTGAAATTGTTG
AAAGGGAAGTGCTTGAGATCAGTCTTAAGGTTTAGTGATCAGTCTTCCTTTTGGTT
GGTGCACTCACTATTTCTTAGGCCAACATCAGTTTTGGCGGCAGGATAATGACATT
GGAATGTGGCTCTTTCGGGAGTGTTATAGCCTTTGTTGATACTGCCTGCTTAGACT
GAGGACTGCGCTTTTGCTAGGA

112. ***Lipomyces starkeyi*** **SWU-NATP 2-6**

>SWU-NATP 2-6 (528 bp)

GCCTTAGTAACTGCGAGTGAAGCGGCAAGAGCTCAAATTTGAAATCTGGCACCTT
CGGTGTCCGAGTTGTAATTTGTAGAAGCATCTTTTGGATTGGTCCTTGCATATGTTC
CTTGGAACAGGACATCATAGAGGGTGAGAATCCCGTTCATGGTGGGGAATCCAA
TTCTTTGTAAAGTGCTTTCGAAGAGTCGAGTTGTTTGGGAATGCAGCTCTAAGTGG
GTGGTAAATTCCATCTAAAGCTAAATATTGGCGAGAGACCGATAGCGAACAAGT
ACAGTGATGGAAAGATGAAAAGAACTCCGAAAGGAGAGTGAAAAAGGACGTGA
AATTGTTGAAAGGGAAGTGCTTGAGATCAGTCTTAAGGTTTAGTGATCAGTCTTCC
TTTTGGTTGGTGCACTCACTATTTCTTAGGCCAACATCAGTTTTGGCGGCAGGATA
ATGGCATTGGAATGTGGCTCTTTCGGGAGTGTTATAGCCTTTGTTGATACTGCCTG
CTTAGACTGAGGACTGCGCTTTTGCTAGGAT

113. ***Lipomyces starkeyi*** **SWU-NAP 8-1**

>SWU-NAP 8-1 (537 bp)

ATTGCCTTAGTAACTGCGAGTGAAGCGGCAAGAGCTCAAATTTGAAATCTGGCAC
CTTCGGTGTCCGAGTTGTAATTTGTAGAAGCATCTTTTGGATTGGTCCTTGCATATG
TTCCTTGGAACAGGACATCATAGAGGGTGAGAATCCCGTTCATGGTGGGGAATCC
AATTCTTTGTAAAGTGCTTTCGAAGAGTCGAGTTGTTTGGGAATGCAGCTCTAAGT
GGGTGGTAAATTCCATCTAAAGCTAAATATTGGCGAGAGACCGATAGCGAACAA
GTACAGTGATGGAAAGATGAAAAGAACTCCGAAAGGAGAGTGAAAAAGGACGT

GAAATTGTTGAAAGGGAAGTGCTTGAGATCAGTCTTAAGGTTTAGTGATCAGTCT
TCCTTTTGGTTGGTGCACTCACTATTTCTTAGGCCAACATCAGTTTTGGCGGCAGG
ATAATGACATTGGAATGTGGCTCTTTCGGGAGTGTTATAGCCTTTGTTGATACTGC
CTGCTTAGACTGAGGACTGCGCTTTTGCTAGGATGTTGGC

114. ***Lipomyces starkeyi*** **SWU-NGTP 4-3**

>SWU-NGTP 4-3 (539 bp)

TGCCTTAGTAACTGCGAGTGAAGCGGCAAGAGCTCAAATTTGAAATCTGGCACCT
TCGGTGTCCGAGTTGTAATTTGTAGAAGCATCTTTTGGATTGGTCCTTGCATATGTT
CCTTGGAACAGGACATCATAGAGGGTGAGAATCCCGTTCATGGTGGGGAATCCA
ATTCTTTGTAAAGTGCTTTCGAAGAGTCGAGTTGTTTGGGAATGCAGCTCTAAGTG
GGTGGTAAATTCCATCTAAAGCTAAATATTGGCGAGAGACCGATAGCGAACAAG
TACAGTGATGGAAAGATGAAAAGAACTCCGAAAGGAGAGTGAAAAAGGACGTG
AAATTGTTGAAAGGGAAGTGCTTGAGATCAGTCTTAAGGTTTAGTGATCAGTCTT
CCTTTTGGTTGGTGCACTCACTATTTCTTAGGCCAACATCAGTTTTGGCGGCAGGA
TAATGGCATTGGAATGTGGCTCTTTCGGGAGTGTTATAGCCTTTGTTGATACTGCC
TGCTTAGACTGAGGACTGCGCTTTTGCTAGGATGTTGGCATAA

115. ***Lipomyces tetrasporus*** **SWU-NAP 4-1**

>SWU-NAP 4-1 (499 bp)

GCTCAATTTGAAATCTGGCACCTTCGGTGTCCGAGTTGTAATTTGTAGAAGCATCT
TTTGGATTGGTCCTTGCATATGTTCCTTGGAACAGGACATCATAGAGGGTGAGAA
TCCCGTTCATGGTGGGGAATCCAATTCTTTGTAAAGTGCTTTCGAAGAGTCGAGTT
GTTTGGGAATGCAGCTCTAAGTGGGTGGTAAATTCCATCTAAAGCTAAATATTGG
CGAGAGACCGATAGCGAACAAGTACAGTGATGGAAAGATGAAAAGAACTCCGA
AAGGAGAGTGAAAAAGGACGTGAAATTGTTGAAAGGGAAGTGCTTGAGATCAGT
CTTAAGGTTTAGTGATCAGTCTTCCTTTTGGTTGGTGCACTCACTATTTCTTAGGCC
AACATCAGTTTTCGCGGCAGGATAATGACATTGGAATGTGGCTCTTTCGGGAGTG
TTATAGCCTTTGTTGATACTGCCTGCTTAGACTGAGGACCGCGCTTTTGCTAGGAT
GT

116. ***Hannaella*** **sp. SWU-NAPS 5-1**

>SWU-NAPS 5-1 (501 bp)

AATTTAAAATCTGGCGTCTTTCAGGCGTCCGAGTTGTAATCTACAGAAGTGTTTTC
CGTGCCGGACCGTGTCCAAGTCCCTTGGAATAGGGTATCAAAGAGGGTGATAATC
CCGTACTTGACACGACAACCGGTGCTCTGTGATACATTTTCTACGAGTCGAGTTGT
TTGGGAATGCAGCTCAAAATGGGTGGTAAATTCCATCTAAAGCTAAATATAGGCG
AGAGACCGATAGCGAACAAGTACCGTGAGGGAAAGATGAAAAGCACTTTGGAA
AGAGAGTTAAACAGTACGTGAAATTGTTAAAAGGGAAACGATTGAAGTCAGTCA
TGTCTATTGGTTTCAGCTAGCTCTGCTAGTGTATTACCTTTAGACGGGTCAACATC
AGTTTTAGACGGTGGAAAAAGGCATGAGGAAAGTAGCAACTCCGGTTGTGTTAT
AGCCTCGTGTTGCATACACTGTCCGAGACTGAGGAATGCAGCTCGCCTTTATGGC
CGGGGTT

117. ***Papiliotrema flavescens*** **SWU-NGTP 2-6**

>SWU-NGTP 2-6 (500 bp)

TGAAATCTGGCGTGCTCAGTGCGTCCGAGTTGTAATCTATAGAGTCGTTTTCCGTG
CCGGACTGTGTCCAAGTCCCTTGGAACAGGGTATCAAAGAGGGTGATAATCCCGT
ACTTGACACAATGACCGGTGCTCTGTGATACGTCTTCTACGAGTCGAGTTGTTTGG
GAATGCAGCTCAAAATGGGTGGTGAGTTCCATCTAAAGCTAAATATTGGCGAGA
GACCGATAGCGAACAAGTACCGTGAGGGAAAGATGAAAAGCACTTTGGAAAGA
GAGTTAAACAGTACGTGAAATTGTTAAAAGGGAAACGATTGAAGTCAGTCGTGA
CTGAGAGGCTCAGCCGGTTCTGCCGGTGTATTCCCCTCAGTCGGGTCAACATCAG
TTTTGTTCGGTGGATAAGGGCAGTTGGAAGGTGGCACCCTCGGGTGTGTTATAGC
CAGCTGTCGCATACATCGGATGAGACTGAGGAATGCAGCTCGCCTTTATGGCCGG
GGTTCGC

118. ***Cyberlindnera saturnus*** **SWU-NGTP 4-12-2**

>SWU-NGTP 4-12-2 (548 bp)

CTCAGTAACGGCGAGTGAAGCGGCAAAAGCTCAAATTTGAAATCTGGTACCTTTG
GTGCCCGAGTTGTAATTTGAAGATAGTTTTCTGGTGCTGGCCCTTGTCTATGTTCCT
TGGAACAGGACGTCACAGAGGGTGAGAATCCCGTCTGGCGGGGTGTCCAGTGCT
TTGTAGATTTCTATCGACGAGTCGAGTTGTTTGGGAATGCAGCTCTAAGTGGGTGG
TAAATTCCATCTAAAGCTAAATATTGGCGAGAGACCGATAGCGAACAAGTACAG
TGATGGAAAGATGAAAAGAACTTTGAAAAGAGAGTGAAAAAGTACGTGAAATT

GTTGAAAGGGAAGGGTATTAGATCAGACTTGGTGTTTTGTGATTATCTTCCCTTCT
TGGGTTGTGCACTCGCATTTCACTGGGCCAGCATCGGTTCGGGTGGTAAGATAAT
GACATTGGAACGTGGCACTACCTTCGGGTGGTGTGTTATAGCCCTTGTTGATGTTG
CCTACCTGGACCGAGGACTGCGGCTTTTGCCTAGGATGCTGGCGTAATGATC

119. ***Cyberlindnera saturnus*** **SWU-NGTP 5-3-3**
>SWU-NGTP 5-3-3 (484 bp)
CTGGTACCTTTGGTGCCCGAGTTGTAATTTGAAGATAGTTTTCTGGTGCTGGCCCTT
GTCTATGTTCCTTGGAACAGGACGTCACAGAGGGTGAGAATCCCGTCTGGCGGGG
TGTCCAGTGCTTTGTAGATTTCTATCGACGAGTCGAGTTGTTTGGGAATGCAGCTC
TAAGTGGGTGGTAAATTCCATCTAAAGCTAAATATTGGCGAGAGACCGATAGCG
AACAAGTACAGTGATGGAAAGATGAAAAGAACTTTGAAAAGAGAGTGAAAAAG
TACGTGAAATTGTTGAAAGGGAAGGGTATTAGATCAGACTTGGTGTTTTGTGATT
ATCTTCCCTTCTTGGGTTGTGCACTCGCATTTCACTGGGCCAGCATCGGTTCGGGT
GGTAAGATAATGACATTGGAACGTGGCACTACCTTCGGGTGGTGTGTTATAGCCC
TTGTTGATGTTGCCTACCTGGACCGAGGACTGCGGCTTTTGCC

120. ***Saitozyma podzolica*** **SWU-NGP 5-3-8**
>SWU-NGP 5-3-8 (496 bp)
TTTGAAAGCTGGCGTCCTACGGGCGTCCGCATTGTAATCTATAGAGGCGTTTTCTG
TGCTGGACCGTGTCCAAGTCCCTTGGAACAGGGTATCAAAGAGGGTGACAATCC
CGTACTTGACACGACAACCAGTGCTCTGTGATACGTCTTCTACGAGTCGAGTTGTT
TGGGAATGCAGCTCAAAATGGGTGGTGAATTCCATCTAAAGCTAAATATTGGCGA
GAGACCGATAGCGAACAAGTACCGTGAGGGAAAGATGAAAAGCACTTTGGAAA
GAGAGTTAAACAGTATGTGAAATTGTTGAAAGGGAAACGATGGAGGTCAGTCGT
GTCTGTGGGATTCAGCCGTCTCTGGCGGTGTACTTCCCACAGACGGGTCAACATC
AGTTTTGGCTGGCGGATAAAGGCAGGAGGAAGGTGGCACCCCCGGGTGTGTTAT
AGCCTCTTGTTGCATACGCTGGCTGAGACTGAGGAATGCAGCTCGCCTTTATGGC
CGGG

121. ***Piskurozyma*** **sp. SWU-NGP 14-3-4**
>SWU-NGP 14-3-4 (583 bp)
TTCCCCTAGTAACGGCGAGTGAAGCGGGAAGAGCTCAAATTTGAAATCTGGCGC
CCTCAGGACGTCCGAGTTGTAATCTATAGGAACGTTTTCCGTGCCGGCTCATGTAC

AAGTCCCTTGGAACAGGGCGTCATAGAGGGTGAGAATCCCGTACTTGACATGAA
CCCCCGGTGCTCTGTGATACGTCTCCAACGAGTCGAGTTGTTTGGGAATGCAGCTC
AAAATGGGTGGTAAATTCCATCTAAAGCTAAATATTGGTGTGAGACCGATAGCG
AACAAGTACCGTGAGGGAAAGATGAAAAGCACTTTGGAAAGAGAGTTAAAAAG
TATGTGAAATTGTTGAAAGGGAAACGATTGAAGTCAGTCGTGTCCTTCAGATTCA
GCTGGTTCTGCCGGTGTATTTCTGTTGGATGGGGTCAACATCAGTTTCGGATGCCG
GATAAGGGCTGGAGGAAGGTAGTACCCCCGGGTAAACTTATAGCCTCCTGTCGC
ATACGGTGACTGGGACTGAGGAACGCAGCTTGCCTTCATGGCCGGGGTTCGCCCA
CGTACAAGCTTAGGATGTTGACATAATGGCTTTAAA

122. ***Papiliotrema flavescen*s SWU-NGTP 4-1**
>SWU-NGTP 4-1 (480 bp)
GAAATCTGGCGTGCTCAGTGCGTCCGAGTTGTAATCTATAGAGTCGTTTTCCGTGC
CGGACTGTGTCCAAGTCCCTTGGAACAGGGTATCAAAGAGGGTGATAATCCCGTA
CTTGACACAATGACCGGTGCTCTGTGATACGTCTTCTACGAGTCGAGTTGTTTGGG
AATGCAGCTCAAAATGGGTGGTGAGTTCCATCTAAAGCTAAATATTGGCGAGAG
ACCGATAGCGAACAAGTACCGTGAGGGAAAGATGAAAAGCACTTTGGAAAGAG
AGTTAAACAGTACGTGAAATTGTTAAAAGGGAAACGATTGAAGTCAGTCGTGAC
TGAGAGGCTCAGCCGGTTCTGCCGGTGTATTCCCCTCAGTCGGGTCAACATCAGTT
TTGTTCGGTGGATAAGGGCAGTTGGAAGGTGGCACCCTCGGGTGTGTTATAGCCA
GCTGTCGCATACATCGGATGAGACTGAGGAATGCAGCTCGC

123. ***Saitozyma podzolica*** **SWU-NGP 14-2-2**
>SWU-NGP 14-2-2 (481 bp)
GGGAAGAGCTCAAATTTGAAAGCTGGCGTCCTACGGGCGTCCGCATTGTAATCTA
TAGAGGCGTTTTCTGTGCTGGACCGTGTCCAAGTCCCTTGGAACAGGGTATCAAA
GAGGGTGACAATCCCGTACTTGACACGACAACCAGTGCTCTGTGATACGTCTTCT
ACGAGTCGAGTTGTTTGGGAATGCAGCTCAAAATGGGTGGTGAATTCCATCTAAA
GCTAAATATTGGCGAGAGACCGATAGCGAACAAGTACCGTGAGGGAAAGATGA
AAAGCACTTTGGAAAGAGAGTTAAACAGTATGTGAAATTGTTGAAAGGGAAACG
ATGGAGGTCAGTCGTGTCTGTGGGATTCAGCCGTCTCGGCGGTGTACTTCCCACA
GACGGGTCAACATCAGTTTTGGCTGGCGGATAAAGGCAGGAGGAAGGTGGCACC
CCCGGGTGTGTTATAGCCTCTTGTTGCATACGCTGGCTGAGACTG

124. ***Piskurozyma*** **sp. SWU-NGP 14-3-2**

>SWU-NGP 14-3-2 (517 bp)

CCTAGTAACGGCGAGTGAAGCGGAAGAGCTCAAATTTGAAATCTGGCGCCCTC
AGGACGTCCGAGTTGTAATCTATAGGAACGTTTTCCGTGCCGGCTCATGTACAAG
TCCCTTGGAACAGGGCGTCATAGAGGGTGAGAATCCCGTACTTGACATGAACCCC
CGGTGCTCTGTGATACGTCTCCAACGAGTCGAGTTGTTTGGGAATGCAGCTCAAA
ATGGGTGGTAAATTCCATCTAAAGCTAAATATTGGTGTGAGACCGATAGCGAACA
AGTACCGTGAGGGAAAGATGAAAAGCACTTTGGAAAGAGAGTTAAAAAGTATGT
GAAATTGTTGAAAGGGAAACGATTGAAGTCAGTCGTGTCCTTCAGATTCAGCTGG
TTCTGCCGGTGTATTTCTGTTGGATGGGGTCAACATCAGTTTCGGATGCCGGATAA
GGGCTGGAGGAAGGTAGTACCCCCGGGTAAACTTATAGCCTCCTGTCGCATACGG
TGACTGGGACTGAGGAACGCAG

125. ***Meyerozyma guilliermondii*** **SWU-NATP 2-4**

>SWU-NATP 2-4 (442 bp)

AGTGAAGCGGCAAAAGCTCAAATTTGAAATCTGGCGCCTTCGGTGTCCGAGTTGT
AATTTGAAGATTGTAACCTTGGGGTTGGCTCTTGTCTATGTTTCTTGGAACAGGAC
GTCACAGAGGGTGAGAATCCCGTGCGATGAGATGCCCAATTCTATGTAAGGTGCT
TTCGAAGAGTCGAGTTGTTTGGGAATGCAGCTCTAAGTGGGTGGTAAATTCCATC
TAAAGCTAAATATTGGCGAGAGACCGATAGCGAACAAGTACAGTGATGGAAAG
ATGAAAAGAACTTTGAAAAGAGAGTGAAAAAGTACGTGAAATTGTTGAAAGGG
AAGGGTTTGAGATCAGACTCGATATTTTGTGAGCCTTGCCTTCGTGGCGGGGTGA
CCCGCAGCTTATCGGGCCAGCATCGGTTTGGGCGGTAGGATAATGGCGTAGGAAT
GTGAC

126. ***Saitozyma podzolica*** **SWU-NGP 14-2-2**

>SWU-NGP 14-2-2 (505 bp)

AGCTCAAATTTGAAAGCTGGCGTCCTACGGGCGTCCGCATTGTAATCTATAGAGG
CGTTTTCTGTGCTGGACCGTGTCCAAGTCCCTTGGAACAGGGTATCAAAGAGGGT
GACAATCCCGTACTTGACACGACAACCAGTGCTCTGTGATACGTCTTCTACGAGT
CGAGTTGTTTGGGAATGCAGCTCAAAATGGGTGGTGAATTCCATCTAAAGCTAAA
TATTGGCGAGAGACCGATAGCGAACAAGTACCGTGAGGGAAAGATGAAAAGCA
CTTTGGAAAGAGAGTTAAACAGTATGTGAAATTGTTGAAAGGGAAACGATGGAG
GTCAGTCGTGTCTGTGGGATTCAGCCGTCTCTGGCGGTGTACTTCCCACAGACGG
GTCAACATCAGTTTTGGCTGGCGGATAAAGGCAGGAGGAAGGTGGCACCCCCGG

GTGTGTTATAGCCTCTTGTTGCATACGCTGGCTGAGACTGAGGAATGCAGCTCGC
CTTTATGGCCGGGG

127. ***Papiliotrema flavescens*** **SWU-NATP 3-3**

>SWU-NATP 3-3 (484 bp)
ATTTGAAATCTGGCGTGCTCAGTGCGTCCGAGTTGTAATCTATAGAGTCGTTTTCC
GTGCCGGACTGTGTCCAAGTCCCTTGGAACAGGGTATCAAAGAGGGTGATAATCC
CGTACTTGACACAATGACCGGTGCTCTGTGATACGTCTTCTACGAGTCGAGTTGTT
TGGGAATGCAGCTCAAAATGGGTGGTGAGTTCCATCTAAAGCTAAATATTGGCGA
GAGACCGATAGCGAACAAGTACCGTGAGGGAAAGATGAAAAGCACTTTGGAAA
GAGAGTTAAACAGTACGTGAAATTGTTAAAAGGGAAACGATTGAAGTCAGTCGT
GACTGAGAGGCTCAGCCGGTTCTGCCGGTGTATTCCCCTCAGTCGGGTCAACATC
AGTTTTGTTCGGTGGATAAGGGCAGTTGGAAGGTGGCACCCTCGGGTGTGTTATA
GCCAGCTGTCGCATACATCGGATGAGACTGAGGAATGCAGCTCGC
